# Si-Based Lithium-Ion Battery Anodes: Material Design and Challenges

**DOI:** 10.3390/ma19122580

**Published:** 2026-06-15

**Authors:** Yuyang Wu, Zhifeng Wang

**Affiliations:** 1 “The Belt and Road Initiative” Advanced Materials International Joint Research Center of Hebei Province, School of Materials Science and Engineering, Hebei University of Technology, Tianjin 300401, China; 2 Arizona College of Technology at Hebei University of Technology, Tianjin 300401, China

**Keywords:** lithium-ion batteries, silicon anode, electrochemical performance, challenge

## Abstract

Lithium-ion batteries with high energy density and long cycle life have been widely used as secondary batteries in electric vehicles and energy storage systems. With the growing demand for high energy density in lithium-ion batteries, silicon-based materials, which possess a high theoretical specific capacity (4200 mAh g^−1^), are regarded as core candidates for anode materials. However, Si-based materials undergo severe volume expansion (up to 300%), which leads to the collapse of the electrode structure, inducing pulverization of the active material and capacity loss, thereby hindering the commercial application of silicon-based materials. To address these issues, scholars from various countries have developed many silicon-based materials with different compositions and three-dimensional structures, and have made some research progress. This review first elaborates on the lithium storage mechanisms and advantages of diverse silicon-based anode materials by taking Si, SiO_x_, SiN_x_, and SiP_x_ as representative examples with distinct characteristics. Subsequently, from the two aspects of dimensional design (0D, 1D, 2D and 3D) and architecture design (core–shell, sandwich-like and network structure), the design strategies for various silicon-based anode structures and their enhancement on electrochemical performance are analyzed. Finally, this review elucidated the challenges faced by silicon-based anodes from the perspectives of mechanism elucidation, structural customization, industrialization, and full-cell applications. It also proposed future development directions for silicon anodes by combining actual challenges and focusing on aspects such as structure optimization, machine learning, advanced characterization techniques, and mechanistic analysis.

## 1. Introduction

Traditional fossil energy sources are at risk of depletion due to their non-renewable nature. Moreover, the combustion of traditional energy sources releases carbon dioxide and harmful pollutants [1,2,3,4,5,6,7]. Therefore, the current global industrial energy transition urgently requires efficient and clean renewable energy sources [8,9,10,11,12,13,14,15]. Lithium-ion batteries (LIBs) have the advantages of efficient energy storage, long cycle life, and low environmental impact, thereby effectively addressing the shortcomings of traditional energy sources [16,17,18,19,20,21,22]. Carbon-based materials are the traditional anode materials for LIBs [23,24,25]. However, the theoretical specific capacity of carbon-based materials is relatively low, which makes them unable to meet the requirements of both common and special application scenarios [26,27,28]. Si-based materials have an extremely high theoretical specific capacity (4200 mAh g^−1^), and their kinetic performance can be improved after modification [29,30]. In this situation, silicon-based materials, which are suitable for scenarios requiring high capacity, fast charging, and low-temperature performance, present extremely high application value [31,32,33] and have received widespread attention [34,35,36].

**Figure 1:**
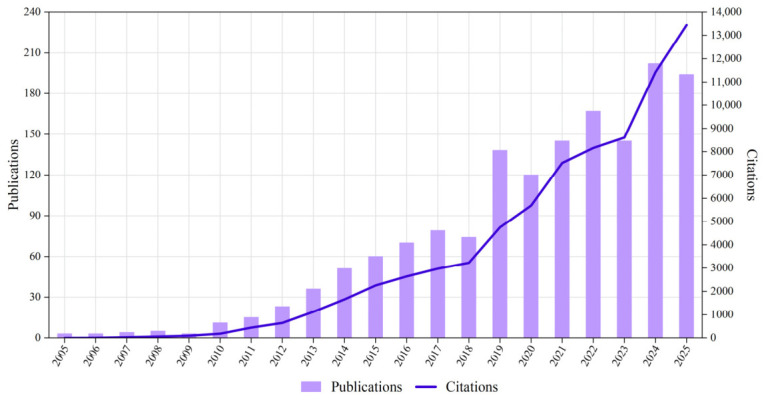
Publications and citations of Si-based anodes for Li-ion batteries (2005~2025, Web of Science platforms).

In recent years, the application of silicon as an anode material for LIBs has garnered significant research attention [37,38,39,40,41,42]. From 2005 to 2025, the annual number of publications and citations concerning Si-based anodes in LIBs shows a clear upward trend (Figure 1). Such continuous increase trends convincingly verify that silicon-based anodes possess substantial application potential for LIBs, thereby making this topic worthy of in-depth exploration within the energy storage community. Usually, there are two main ways to optimize the electrochemical performance of anodes: structural design and developing novel silicon-based materials [43,44,45,46,47]. Accordingly, numerous comprehensive reviews have been published to systematically summarize these research efforts. Zhu et al. reviewed the microscale design of Si-based anodes with densified microstructure [48]. Based on this foundation, the current review supplements the various Si-based materials in the nanoscale with different dimensional structures (0D, 1D, 2D and 3D). In addition to introducing Si materials, more active materials such as SiO_x_, SiN_x_ and SiP_x_ are also introduced in the current work. Zhang et al. summarized the structural evolution of silicon anodes and focused on analyzing the core–shell structure and yolk-shell structure [49]. Based on this, the current review further discusses the architecture structures of silicon anodes, including sandwich-like structures and network structures. Li et al. compared different dimensional structural designs of silicon materials, systematically summarized the composite strategies of Si with various materials and pointed out that Si/C composite materials are the closest to commercialization silicon materials [50]. To enhance the persuasiveness of the best commercial candidates, the current review further evaluated the market progress of different silicon materials and structures from aspects of cost, safety, scalability and electrochemical performance. The review reported by Zhao et al. discussed the roles of structure and interface engineering design by analyzing the electrochemical performance of the various materials. The review also analyzed the principles and applications of various in situ characterization techniques in LIBs [51]. The current review further summarizes the combined use of various in situ characterization methods and analyzes the significant role of this strategy in revealing the dynamic changes in structure and failure mechanisms. Therefore, the current review is necessary as it provides a more comprehensive framework for the design of silicon-based anodes, covering dimensional structure, architectural structure, and various materials. It also combines structural design with the full-cell performance and market readiness, offering clearer guidance for promoting commercialization.

To provide valuable insights for the practical application of high-energy-density LIBs in the future, this review comprehensively summarizes various silicon-based anode materials, the structure design of silicon anodes, and the challenges faced in future development. Firstly, this review elaborates on the unique advantages of different silicon-based materials in LIBs and further analyzes their lithium storage mechanism and electrochemical performance. Subsequently, the structure of the silicon anode is analyzed in detail, reviewing the current research status of dimensional design strategies, including 0D, 1D, 2D and 3D structure designs. Discussions on architecture design are also carried out, with a focus on the core–shell structure, sandwich-like structure and network structure. Finally, this review also elaborates on the challenges faced by silicon-based anodes and presents forward-looking insights. This work provides valuable references for the design and development of silicon-based anodes and the promotion of their application as high-performance anode materials for LIBs.

## 2. Diverse Si-Based Anode Materials

Si-based materials, including silicon, silicon oxide, silicon nitride, and silicon phosphide, have surpassed the theoretical lithium storage capacity of graphite anodes and have thus become the core research and development direction for high-energy-density anode materials of LIBs. The differences in chemical composition and bonding structure result in different volume expansion rates, electron/ion transport efficiency, and lithium storage mechanisms for various Si-based materials. This section focuses on the analysis of the lithium storage mechanism, unique structure design, and electrochemical performance of different electrodes.

### 2.1. Silicon

Among various anode materials for LIBs, silicon is regarded as one of the most attractive options. Silicon has a relatively high abundance in the Earth’s crust and is widely available. Moreover, silicon possesses a high melting point and excellent thermal stability. As a highly competitive candidate for LIB anodes, the main advantages of silicon are as follows: (1) Compared with the interlayer insertion mechanism of graphite, the alloying lithium storage mechanism of silicon endows it with an extremely high theoretical specific capacity. (2) The low operating potential of the silicon anode enhances the operating voltage and energy density of the full-cell. (3) Silicon has a high volumetric specific capacity, making it suitable for high volumetric energy density. However, the development of Si anodes is fraught with challenges, which include (1) the large volume variation during lithiation/delithiation processes, (2) the susceptibility of silicon particles to cracking and pulverization, (3) the performance degradation of active materials, (4) the high instability and continuous regeneration of the SEI, and (5) the relatively low initial Coulombic efficiency and intrinsic electronic conductivity. Although silicon-based anode materials still face many challenges in practical applications, their extremely high theoretical specific capacity is a prominent advantage sufficient to offset the impact of the existing deficiencies. Silicon materials have become a research hotspot in the field of LIB anodes, and have demonstrated significant research value and application prospects in both academic research and industrial applications.

Clarifying the lithium storage mechanism of silicon is a crucial prerequisite for enhancing the structural stability and electrochemical performance. The lithium storage mechanism of silicon materials involves the conversion between crystalline silicon (c-Si) and amorphous silicon (a-Si), as well as the formation of various Li-Si binary intermediate phases. Wen et al. [52] revealed that the four intermediate phases of this binary system at high temperatures are Li_12_Si_7_, Li_7_Si_3_, Li_13_Si_4_, and Li_22_Si_5_. Obrovac et al. [53] demonstrated that at room temperature, c-Si first transforms into a-Li_x_Si during lithiation, and the a-Li_x_Si with a high lithium content subsequently crystallizes into the Li_15_Si_4_ crystal phase. Wang et al. [54] revealed that through in situ TEM tests, the lithiation of a-Si follows a two-phase mechanism, with a clear amorphous-amorphous phase boundary separating the unreacted amorphous silicon from the lithiation product a-Li_x_Si.

The initial discharge process can be stated as:

(1) $$\mathrm{Si}(c)+{x\mathrm{Li}}^{+}+{xe}^{-}\to {\mathrm{Li}}_{x}Si(a)+\left(3.75-x\right){\mathrm{Li}}^{+}+\left(3.75-x\right)e^{-}\to {\mathrm{Li}}_{15}{Si}_{4}\left(c\right)$$

The charging process is as follows:

(2) $${\mathrm{Li}}_{15}{Si}_{4}\left(c\right)\to 15{\mathrm{Li}}^{+}+15e^{-}+4\mathrm{Si}\left(a\right)$$

The following discharge process can be described as:

(3) $$\mathrm{Si}\left(a\right)+{x\mathrm{Li}}^{+}+{xe}^{-}\to {\mathrm{Li}}_{x}Si\left(a\right)+\left(3.75-x\right){\mathrm{Li}}^{+}+\left(3.75-x\right)e^{-}\to {\mathrm{Li}}_{15}{Si}_{4}\left(c\right)$$

During the (de)lithiation process, the silicon anode undergoes significant volume changes, and its volume evolution behavior is closely related to the lithium content. To address this issue, Levitas and Attariani [55] established a quantitative relationship between the volume change in amorphous Li_x_Si and the lithium content *x*, which further reflected the inconsistency in the volume evolution of the material during the (de)lithiation process.

In the process of lithiation:

(4) $$\varepsilon_{0}^{l}=0.01\left(12.37x^{4}-58.72x^{3}+100x^{2}+3.326x\right)$$

During the delithiation:

(5) $$\varepsilon_{0}^{dl}=0.01\left(1.037x^{4}-12.97x^{3}+31.04x^{2}+44.27x+9.187\right)$$

where $\varepsilon_{0}^{l}$ is the volumetric strain during lithiation, and $\varepsilon_{0}^{dl}$ is the volumetric strain during delithiation.

In silicon-based anodes, the migration of lithium mainly takes two forms: bulk diffusion and interfacial diffusion. Bulk diffusion involves the migration process of lithium ions within the silicon material (crystalline or amorphous). Tritsaris et al. used first-principles density functional theory to uncover that the migration energy barrier of lithium in crystalline silicon is 0.55 eV, while the migration energy barrier of lithium in amorphous silicon ranges from 0.1 to 2.4 eV, with an average single migration energy barrier of 0.58 eV [56]. Interface diffusion mainly encompasses the process where lithium ions pass through the SEI and into the surface of silicon particles, as well as the process where lithium ions traverse the interface between c-Si and a-Li_x_Si. These processes typically have high energy barriers and determine the rate performance of silicon materials. Ramasubramanian et al. focused on analyzing the migration energy barriers of lithium in the three main components of the SEI, namely Li_2_O, LiF, and Li_2_CO_3_. It is proven that the migration energy barrier of lithium at the grain boundaries formed between these components is significantly lower than that within the component grains. And the migration energy barrier of the dense grain boundary is the largest, at 1.03 eV [57]. Lithium insertion into crystalline silicon will lead to the formation of a-Li_x_Si, which in turn results in the generation of the c-Si and a-Li_x_Si interfaces. Li et al. demonstrated that due to the significant lattice distortion at the interface between c-Si and a-Li_x_Si, the migration energy barrier of lithium at this interface is extremely high [58]. Therefore, the migration process of lithium at the interface between c-Si and a-Li_x_Si is regarded as the key step determining the rate of lithiation.

Nanostructures occupy a central position in the design of silicon anode structures. One-dimensional silicon nanowires, owing to their unique ability to buffer volume expansion and facilitate rapid ion transport, are widely utilized in silicon anodes. Chan et al. [59] first revealed that silicon nanowires can alleviate huge volume expansion through nanoscale effects and their one-dimensional structure, laying a theoretical foundation for the subsequent design of silicon anode nanostructures. The semiconductor properties of silicon result in poor intrinsic electronic conductivity. Therefore, substantial efforts have been devoted to optimizing the conductivity of silicon-based materials. Among these attempts, it is a popular design that utilizes Si-C composite materials to form a continuous conductive framework, thereby constructing a three-dimensional electronic transport network. Yan et al. [60] prepared a material using carbon nanofibers as the conductive framework, achieving a charge transfer resistance of only 131.3 Ω. Metal composite modification is another important strategy for enhancing the electronic conductivity of silicon anodes. Transition metals such as Cu and Ni possess high free electron concentrations and excellent intrinsic metallic conductivity. Hong et al. [61] prepared Cu/Si NWs. When the current density was increased to 2000 mA g^−1^, the reversible capacity of this electrode still reached 1280 mAh g^−1^, with a charge transfer resistance of only 70 Ω.

Through the optimization of structure and conductivity, silicon-based anode materials have achieved considerable commercial application. In commercial LIBs, the mainstream silicon-based anodes are Si/C composite materials. BTR New Material Group Co., Ltd. (Shenzhen, China) is the first enterprise in China to achieve industrial production of silicon-carbon anodes. For the hard carbon-based silicon-carbon anode used in the blade battery of BYD (Shenzhen, China), the silicon content is 18%, and the specific capacity can reach 1600 mAh g^−1^. BASF Shanshan Battery Materials Co., Ltd. (Changsha, China) has adopted the gas-phase nano crystallization and fluidized bed coating processes to achieve mass production of silicon-based materials on a ten-thousand-ton scale. The AS6BP series of silicon-carbon anodes, as the first high-silicon material applied in mobile phones, have a silicon content of 25%. To date, extensive research has been conducted on the lithium storage mechanism and structural design of silicon-based anodes and various morphologies and structures of silicon-based electrode materials have been successfully developed. The relevant progress is systematically elaborated in the subsequent sections. Owing to their extremely high theoretical capacity, silicon-based materials have become the key anode material for next-generation high-energy-density LIBs and possess irreplaceable research value and application status.

### 2.2. Silicon Oxide

Silicon oxides include stoichiometric silicon oxides (SiO_2_, Si_2_O_3_, Si_3_O_4_) and non-stoichiometric silicon oxides SiO_x_ (0 < x < 2), where the latter mainly consist of SiO. Stoichiometric silicon oxides exhibit poor conductivity and a low theoretical capacity (<1000 mAh g^−1^), and are only used as precursor materials for coating or doping with silicon-based anodes. The lithium storage mechanism of non-stoichiometric silicon oxides proceeds via a two-step reaction. The first step occurs during the lithiation process, where oxygen in the oxide undergoes an irreversible reduction reaction with Li^+^, generating inert substances such as Li_2_O and silicates. The second step takes place simultaneously with the reduction reaction, during which free Si is released and achieves lithium storage through the reversible alloying reaction with Li^+^ (where Si and Li form a Li-Si alloy). Accordingly, the theoretical capacity of SiO_x_, represented by SiO, is approximately 1600 mAh g^−1^, making it one of the typical representatives of silicon-based anode materials for commercial applications. The advantages of silicon oxide include higher reversibility than graphite and a significantly lower volume expansion rate than pure silicon. The disadvantages mainly lie in two aspects. First, during the first cycle, Li^+^ consumption caused by the formation of inert matrixes results in a relatively low initial Coulomb efficiency. Second, the conductivity is poor and the lithium diffusion kinetics are weak. To comprehensively improve the performance of silicon oxide as an anode active material, extensive theoretical analysis and practical applications has been conducted.

SiO is a commonly used active material for silicon anodes with significantly enhanced structure and interface stability. Hwa et al. modified SiO through disproportionation reactions to prepare an anode material with excellent electrochemical performance. The electrode exhibits a considerable reversible capacity (1000 mAh g^−1^) and excellent cycle stability [62]. Lee et al. prepared a SiO particle composite with a carbon coating and 3D porous structure. The as-prepared anode exhibits a high specific capacity of up to 1520 mAh g^−1^ and excellent cycling stability, with a reversible capacity remaining at 1490 mAh g^−1^ after 50 cycles [63].

**Figure 2:**
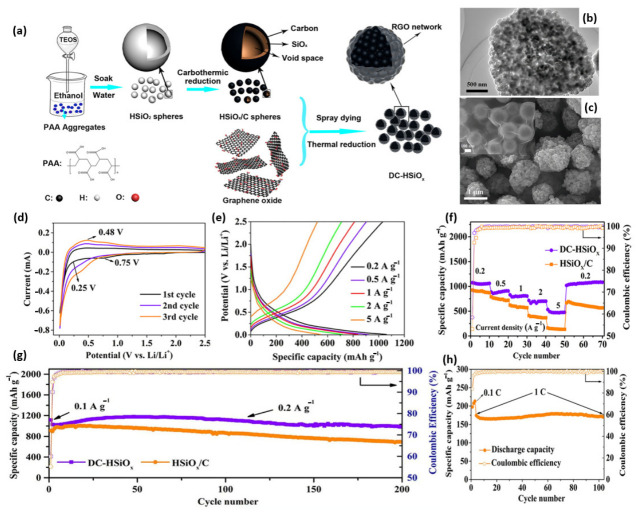
Preparation and electrochemical performance of silicon oxide (DC−HSiO_x_): (**a**) Schematic diagram showing the synthesis procedure. (**b**) TEM image. (**c**) SEM image incorporating an enlarged SEM image as an inset. (**d**) CV curves for the first three cycles. (**e**) Galvanostatic charge/discharge curves at different current densities. (**f**) Rate capabilities of DC−HSiO_x_ and HSiO_x_/C. (**g**) Cycling stability of HSiO_x_/C vs. DC−HSiO_x_ electrodes. (**h**) Electrochemical cycling stability of DC−HSiO_x_//NCM811 full-cell. (Reproduced from [64] with permission from ACS, 2019).

In addition to the common SiO, there are many studies conducted on non-chemically stoichiometric silicon oxides SiO_x_. Xu et al. [64] prepared a hollow SiO_x_ encapsulated within a dual-carbon conductive network as illustrated in Figure 2a. In the DC−HSiO_x_ composite, the average valence state of Si is 2.44, corresponding to x ≈ 1.22. This average silicon valence indicates a well-balanced silicon-oxygen ratio in the SiO_x_ structure. Such an optimized silicon-oxygen ratio allows the material to retain sufficient electrochemically active silicon, while simultaneously generating an appropriate amount of Li_2_O and lithium silicate buffer phases during the initial lithiation process. The sufficient active silicon enables the material to achieve a high reversible lithium storage capacity, and the presence of an appropriate amount of buffer phases can effectively alleviate volume expansion. Therefore, SiO_x_ with this 2.44 average valence state can achieve an excellent balance between capacity and stability. Figure 2b,c illustrates that the as-prepared material exhibits a spherical morphology. The gradual increase in peak intensity of the cathode and anode peaks during the cycling process proves that the materials have a distinct activation process (Figure 2d). Figure 2e,g further demonstrates the excellent reversibility and cycling stability of the materials, which can be attributed to the synergistic effects of the hollow structure and the dual carbon network in mitigating the intrinsic defects of SiO_x_, including severe volume expansion and poor electrical conductivity. The hollow structure effectively buffers (de)lithiation-induced volume changes by reserving cavities within the particles. The amorphous carbon coating layer in the dual carbon conductive network and the reduced graphene oxide conductive framework can significantly reduce the electrode resistance and improve the electron/ion transport rate, thereby enhancing the conductivity and rate performance of the electrode material. Furthermore, in different silicon material designs, the hollow structure typically has a thinner shell layer and a higher specific surface area, facilitating the entry of lithium ions into the active material interior. The dual carbon conductive network often achieves continuous conductive channels and inter-particle conductive connections simultaneously. Figure 2f shows that the materials have excellent rate performance. As shown in Figure 2h, the capacity decay rate of as-prepared materials is only 0.04% per cycle, which verifies the advantages of SiO_x_ over pure Si in terms of higher cycling stability and lower capacity decay. This is because during the initial lithiation process, SiO_x_ forms Li_2_O and lithium silicate, which are uniformly distributed near the nano-silicon, dispersing stress and limiting the growth and agglomeration of active silicon particles, thereby effectively alleviating the drastic volume changes of silicon and improving the electrochemical performance of the material.

These studies, along with the supportive findings in reference [65,66], all indicate that silicon oxide is a highly promising anode active material in the field of lithium-ion batteries. Its core advantage lies in its high theoretical specific capacity. Moreover, compared with pure silicon anodes, silicon oxide exhibits a weaker volume expansion effect during charge and discharge cycles, which can enhance the structural stability and safety of the battery to a certain extent.

### 2.3. Silicon Nitride

Silicon nitrides include stoichiometric silicon nitride (Si_3_N_4_) and non-stoichiometric silicon nitride (SiN_x_, without a fixed Si/N ratio). Crystalline Si_3_N_4_ possesses extremely high mechanical strength but exhibits very low conductivity and a dense lattice, making Li^+^ diffusion extremely difficult. Amorphous Si_3_N_4_ also has high mechanical strength and contains voids in the lattice, allowing slow Li^+^ diffusion. Thus, it is often used as a coating layer for silicon-based particles. Non-stoichiometric silicon nitrides are typically amorphous structures with high mechanical strength, chemical inertness, and moderate ionic conductivity, making them ideal choices for interface coating and structural modification of silicon-based anodes. Unlike the single alloying process of pure silicon, as well as the partial conversion and alloying process of silicon oxides, silicon nitride follows a two-step synergistic lithium storage mechanism. The first step involves conversion, forming Li_3_N, which limits volume expansion by establishing a rigid framework. In the second alloying step, free Si reacts with Li^+^ to achieve charge storage. Additionally, the overall expansion rate of silicon nitride (100~180%) is much lower than that of pure silicon and silicon oxides. In the following paragraphs, several silicon nitrides are taken as examples to elaborate on the related research in detail.

Si_3_N_4_ is a typical material in silicon nitrides. The cycle stability of Si_3_N_4_ is relatively high, but its theoretical lithium storage capacity is relatively low. Sharma et al. selected Si_3_N_4_ as the anode material for all-solid-state lithium-ion batteries. Electrochemical cycling tests showed that the material exhibited excellent capacity retention and high Coulombic efficiency during long-term cycling [67]. Compared with Si_3_N_4_, non-stoichiometric silicon nitride SiN_0.92_ has a lower nitrogen content and a higher silicon content. Therefore, during the lithium storage process, the proportion of silicon atoms released through the breakage of the silicon-nitrogen covalent bond is relatively high, and these silicon atoms do not undergo further chemical combination with or fixation by nitrogen atoms. This characteristic endows SiN_0.92_ with a higher theoretical lithium storage capacity. Suzuki et al. fabricated SiN_0.92_ films using the pulsed laser deposition (PLD) method. The obtained films not only exhibited high capacity values at relatively low potentials (lower than 0.5 V relative to Li^+^/Li) but also showed minimal capacity decay during the cycling [68].

**Figure 3:**
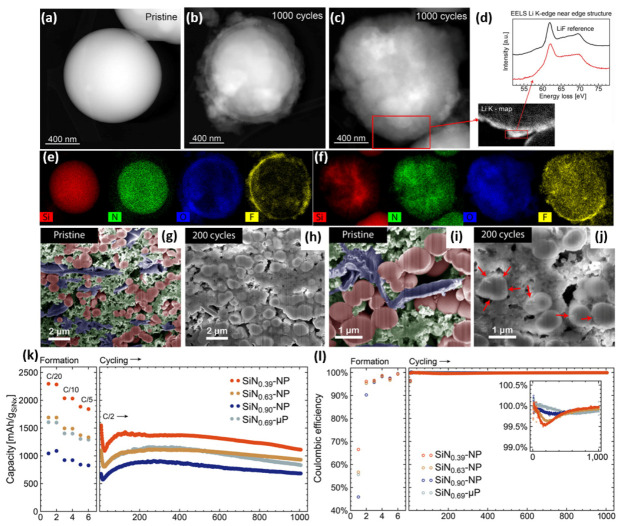
Morphologies and electrochemical performance of SiN_0.69_-μP: (**a**–**c**) TEM images. (**d**) Lithium elemental mapping by EELS and near-edge spectral features of the SEI obtained from EELS. (**e**,**f**) EDS results, where (**e**)corresponds to (**b**), while (**f**)corresponds to (**c**). (**g**–**j**) SEM images. Different components in the electrode are distinguished by false color in the images, where red corresponds to SiN_0.69_-μP, blue corresponds to graphite, and green corresponds to binder. (**k**) Cycling performance and (**l**) Coulombic efficiency of half cells. As reflected in the insert, CE maintains a value near 100% (reproduced from [69] with permission from ACS, 2021).

Controlling the Si/N ratio is an effective measure to balance cycling stability and high specific capacity. Increasing the Si content can enhance the active lithium storage sites and specific capacity, but it will aggravate volume expansion and interface instability. Increasing the N content can increase the nitrogen-containing buffer matrix formed after the initial lithiation and effectively disperse the active Si to avoid agglomeration. However, the increase in N content will lead to a decrease in the content of active Si, thereby reducing the reversible capacity. Therefore, finding the optimal balance point between high capacity and high stability is a key strategy in the design of SiN_x_ materials. Ulvestad et al. [69] prepared a nanostructured conversion-type anode material SiN_x_ (SiN_0.69_-μP, with a stoichiometric ratio of x = 0.69). Figure 3a–f shows that most of the SiN_0.69_-μP maintains its particle morphology integrity and uniform element distribution after long-term cycling, demonstrating its excellent structural stability. This is attributed to the in situ reaction products formed after the initial lithiation of silicon nitride. This can be attributed to the in situ reaction products represented by Li_2_SiN_2_ formed during the initial lithiation of silicon nitride. These in situ reaction products act as an inert matrix, encapsulated around the active Si, which inhibits the volume expansion of the active Si and maintains the structural integrity of the electrode. Additionally, the inert matrix increases the interfacial energy barrier, thereby preventing Si from being deeply lithiated to the extent of forming Li_15_Si_4_, reducing the lithium concentration and inhibiting the growth of Li_15_Si_4_. By comparing the FIB-SEM images before and after cycling, it can be observed that the electrode undergoes enhanced densification during the cycling process, especially in materials with low nitrogen content (Figure 3g–j). This is because SiN_x_ materials with a high nitrogen content generate a larger amount of inert Li_2_SiN_2_ matrix during the initial lithiation process, enabling the active Si to be more fully encapsulated within the inert matrix, thereby effectively suppressing volume expansion. Further, the decrease in the degree of Si volume expansion effectively retains the pore space in the electrode, increasing the porosity, and ultimately improving the densification phenomenon of the electrode. Figure 3k,l proves that SiN_x_ materials have extremely long cycling stability, high capacity retention rate, and extremely high and stable Coulomb efficiency in the later cycles. These studies, along with the findings from [70], indicate that silicon nitride is an ideal anode material for lithium-ion batteries.

### 2.4. Silicon Phosphide

Silicon phosphides include stoichiometric silicon phosphides (mainly SiP and SiP_2_) and non-stoichiometric silicon phosphides (SiP_x_). Stoichiometric silicon phosphides possess lithium storage activity, and their lithium storage mechanism involves a synergy of alloying and intercalation mechanisms; thus, they are often used as active materials for silicon-based anodes. Non-stoichiometric silicon phosphides exhibit higher mechanical strength at high phosphorus content and are therefore frequently used as coating layers. When the silicon content is high, they possess higher lithium storage activity and are often employed as anode active materials. The lithium storage mechanism of silicon phosphide consists of three main steps. First, Li^+^ diffuses into silicon phosphide, triggering gradual breakage of the covalent bonds between Si and P. Second, the cleaved Si combines with Li^+^ to form a Li-Si alloy, while P atoms react with Li^+^ to form Li_3_P. Third, during the delithiation process, the Li-Si alloy and Li_3_P undergo reversible decomposition, releasing Li^+^ and thus completing the (de)lithiation cycle. The core advantage of silicon phosphide lies in its high theoretical specific capacity (2000–3500 mAh g^−1^), which can be achieved without a high silicon content ratio. Furthermore, the low Si–P bond energy, the relatively low Li^+^ insertion resistance, and the ion transport channels derived from Li_3_P all contribute to the significantly higher electronic conductivity of silicon phosphides compared to other silicon-based anode materials. The structural and lithium storage properties of two typical materials, SiP and SiP_2_, are further elucidated below.

SiP has relatively high theoretical lithium storage capacity and electronic conductivity, and its performance is superior to that of pure Si due to the synergistic effect of Si and P elements. Reinhold et al. fabricated SiP as a Si-based anode material, achieving a high specific capacity of approximately 3000 mAh g^−1^, which is highly attractive. The as-prepared material was synthesized via a vapor transport route, yielding a fluffy and flocculent product with a layered 2D crystal micro-strip structure. This structure is expected to facilitate the rapid Li^+^ insertion and diffusion kinetics [71].

**Figure 4:**
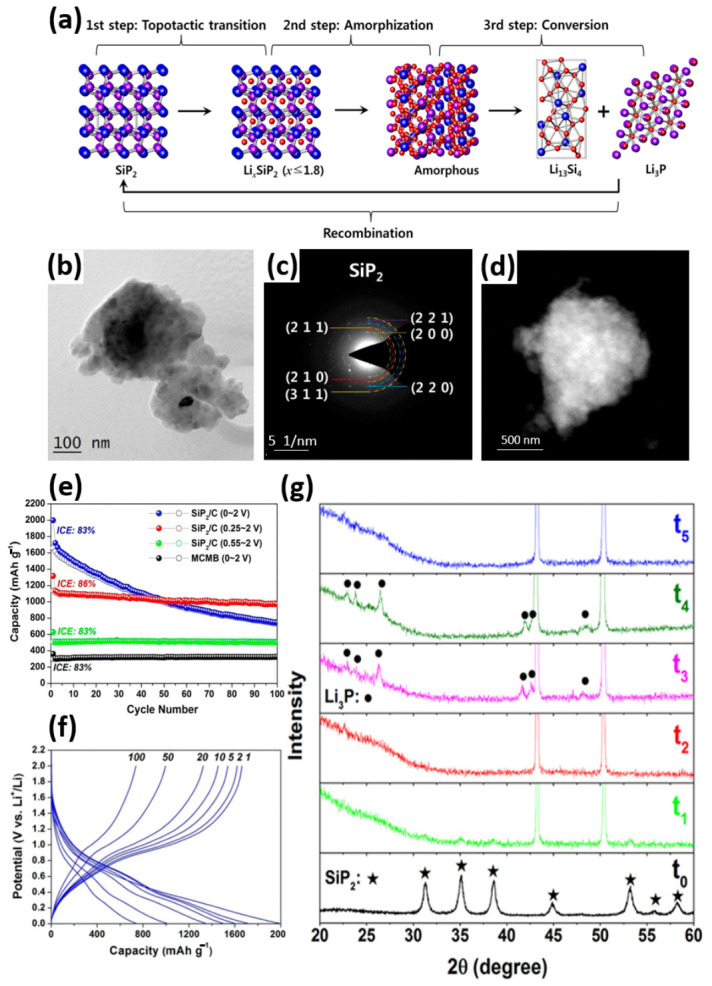
Morphologies and electrochemical performance of SiP_2_/C: (**a**) Schematic illustration of the electrochemical reaction mechanism for the SiP_2_-Li system. (**b**) TEM bright-field image. (**c**) SAED patterns. (**d**) STEM image. (**e**) The cycling stability across different potential windows. (**f**) Voltage profile. (**g**) Ex situ XRD patterns collected in the initial cycle. (Reproduced from [72] with permission from ACS, 2016).

SiP_2_ is another highly promising anode material among silicon phosphides. Compared with SiP, SiP_2_ includes a higher P content, which provides more active sites for lithium storage reactions and endows it with a higher theoretical lithium storage capacity. Kwon et al. [72] prepared a SiP_2_/C composite with a 3D crystalline framework in the cubic crystal system. Figure 4a illustrates the electrochemical reaction mechanism between SiP_2_ and Li. As shown in Figure 4b–d, SiP_2_ nanocrystals are embedded and uniformly dispersed in the amorphous carbon matrix. Figure 4e presents the cycling performance of the SiP_2_/C composite electrode, indicating a capacity retention rate of 98.8% after 100 cycles. As shown in Figure 4f, the SiP_2_/C composite electrode exhibits a high Coulombic efficiency. The excellent electrochemical performance of the as-prepared material is attributed to the mechanically robust 3D crystalline framework of SiP_2_, which effectively shortens the Li^+^ diffusion path, improves the rate performance, and inhibits structural collapse. Furthermore, both Si and P are Li active elements, contributing to the higher theoretical capacity of SiP_2_. In situ XRD results indicate that the phase transition of SiP_2_ during lithium insertion/deinsertion is not a simple alloying reaction, but rather a complex mechanism involving topological embedding, amorphization, and transformation (Figure 4g).

These studies and the related supportive findings [73,74] confirm that silicon phosphides have significant advantages, such as high theoretical specific capacity and superior electronic conductivity compared to other silicon-based anode materials. Owing to the fact that both Si and P can react with lithium and jointly contribute to the lithium storage capacity, silicon phosphides have a much better specific capacity. In terms of conductivity, the Li_3_P formed during the initial lithiation is conducive to the migration of Li^+^. Therefore, silicon phosphides exhibit superior ionic/electrochemical transport properties. However, compared to N and O, P has a larger volume change in lithium storage, resulting in poorer structural stability. In practical applications, by adjusting the ratios of Si/O, Si/N and Si/P, the material performance can be balancedly enhanced. For silicon oxides and silicon nitrides, generally, a lower content of O or N can increase the proportion of active Si and thereby enhance the capacity, while a higher content of O or N can improve the structural stability. Silicon phosphides with an appropriate Si/P ratio can better achieve a balance between capacity, conductivity and cycling stability. In order to facilitate the comparison of the performance of diverse Si-based anode materials, the electrochemical performance and volumetric stability of each of the aforementioned types of Si-based anode materials are summarized, as shown in Table 1. In terms of electrochemical performance, silicon and silicon phosphide exhibit higher capacity compared to silicon oxide and silicon nitride. This is because these two materials contain a higher content of lithium storage active components. In terms of volume stability, pure silicon materials typically exhibit significant volume expansion. Silicon oxides and silicon nitrides usually show relatively lower expansion and better structural stability, which is attributed to the formation of buffer matrix phases. These buffer matrix phases help to accommodate the strain caused by the alloying. Silicon phosphides can provide a high reversible capacity and ICE, but their volume expansion is also relatively significant. Since both Si and P are regarded as high-capacity lithium storage components, the products formed after lithiation have a relatively high volume expansion rate. Moreover, during the lithiation process, there are intermediate products with ion-insulating properties, which lead to uneven local reactions and exacerbate stress concentration. Therefore, their practical application requires the design of nanostructures and conductive/buffer frameworks to alleviate structural degradation. Overall, transitioning from pure silicon to oxides and nitrides reduces volume expansion at a moderate cost to capacity, while phosphides offer high capacity potential but require effective engineering to mitigate extreme volumetric changes.

## 3. Structural Design of Si-Based Materials

Structural design is an effective strategy to alleviate the volume expansion of silicon-based anodes, enhance their electrochemical performance, and achieve long cycle life. Currently, the primary directions in the structural design of silicon-based anodes include dimensional structure design and architectural structure design. Through these two approaches, significant progress has been made toward the practical application of high-energy lithium-ion batteries.

### 3.1. Dimensional Design

The dimensional structure design for silicon anodes focuses on 0D structure (nanoparticles), 1D structure (nanowires, nanotubes and nanofibers), 2D structure (nanosheets), and 3D structure (network structure and composite structure). By leveraging the unique advantages of different dimensional structures in alleviating volume expansion, enhancing electrical conductivity, and improving the stability of the electrode structure, the practical application of silicon anodes has been significantly advanced.

#### 3.1.1. 0D Structure Design

To address the core issues of traditional Si-based anodes, such as high volume expansion and short cycle life, a design strategy focusing on the basic structural unit has been employed to regulate 0D structures, including silicon nanoparticles (SiNPs), at the atomic scale. This design approach initially focused on investigating the intrinsic properties of monodisperse SiNPs. Subsequently, through rational interface modification and dispersion processes, SiNPs were systematically integrated into the electrode system. Ultimately, a Si-based anode material with high capacity and long-term cycling stability was designed. These design strategies significantly support the research and development of 0D Si-based anodes. The following sections elaborate on the characteristics of the two typical structures: the core–shell structure and the nanoparticle structure.

In the 0D structure, the core–shell structure and the nanoparticle structure are two typical representatives. The coating design of the core–shell structure can effectively suppress the severe volume expansion of silicon and isolate the core from the electrolyte. Yi et al. designed a hard carbon/silicon composite material featuring a core–shell structure with a large number of macropores [79]. Chen et al. successfully prepared a composite material (Si@NC) consisting of SiNPs coated with nitrogen-doped porous carbon by calcining the SiNPs encapsulated in the covalent organic framework (COF) [80]. This highly elastic structure with an ordered pore arrangement provides sufficient space for the volume expansion of silicon [80]. The micro-scale design of silicon nanoparticles can disperse the stress caused by volume expansion, thereby reducing particle fragmentation. Sreenarayanan et al. designed a spherical Si-C composite material resembling a pineapple fruit by embedding crystalline nanoparticles into an amorphous carbon matrix. This structure minimizes the loss of total lithium content and enables high-silicon content packing [81].

**Figure 5:**
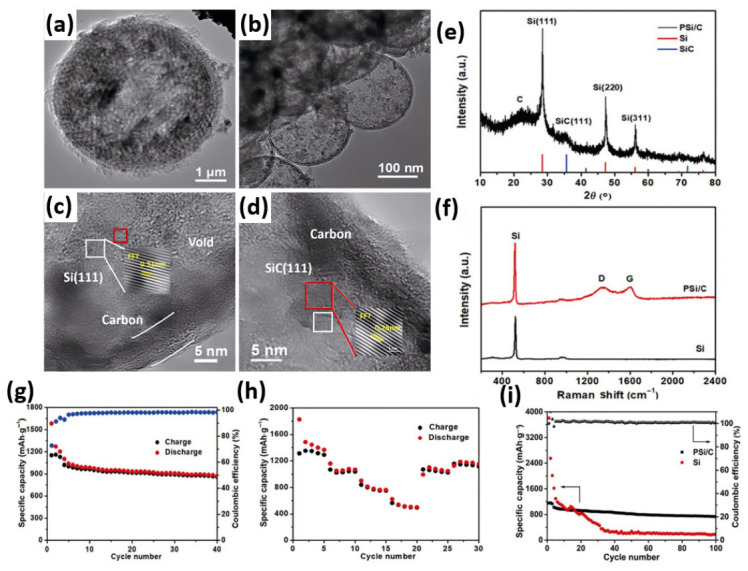
Morphologies and electrochemical performance of 0D structure material (PSi/C): (**a**,**b**) TEM images. (**c**,**d**) HRTEM micrographs where the red area corresponds to SiC and the white area corresponds to Si. (**e**) XRD pattern. (**f**) Raman spectra. (**g**) Cycling performance under a current density of 0.2 A g^−1^. (**h**) Rate performance in different current densities. (**i**) Cycle performance at 1 A g^−1^. (Reproduced from [82] with permission from Tsinghua University Press, 2021).

In the aforementioned 0D structure, the core–shell structure and nanoparticle structure have alleviated the volume expansion issue of silicon and improved battery performance through encapsulation design and size optimization. Wu et al. [82] further prepared a pomegranate-type Si/C composite anode. As shown in Figure 5a–d, the porous Si/C particles are uniformly distributed and well dispersed, which facilitates stress homogenization. The as-prepared material contains amorphous carbon and SiC formed by the direct reaction of silicon with porous carbon (Figure 5e). The presence of amorphous carbon and SiC leads to a dispersed particle structure. As illustrated in Figure 5f, the silicon component in the composite exhibits a fine nanoscale morphology. Compared with micrometer-sized silicon particles, nanosized silicon particles can effectively shorten the lithium-ion diffusion path and improve discharge performance. Figure 5g,i indicates that pomegranate-type particles endow the composite material with excellent cycling performance under both low and high current densities. Furthermore, the composite exhibits excellent rate capability (Figure 5h). These results strongly suggest that 0D particle structure can reduce charge transfer resistance and regulate the formation of a uniform solid electrolyte interphase (SEI) film, thereby enhancing electrochemical performance.

In the research on the design of 0D silicon-based anode structures, extensive exploratory work has continuously improved the structure and enhanced cycling capacity and rate performance of silicon-based anodes [83,84,85]. These studies emphasize that the 0D structure design is an important fundamental solution for the practical application of silicon-based anodes. Although the 0D structure effectively alleviates the issue of local volume expansion, dispersed 0D nanostructures are prone to agglomeration. In contrast, the 1D structure can form a continuous conductive network through directional growth or assembly, connecting the dispersed 0D units in series, thereby addressing the agglomeration problem.

#### 3.1.2. 1D Structure Design

1D structure design, such as nanowires, nanotubes and nanofibers, is a commonly employed strategy in the structural engineering of silicon anodes for lithium-ion batteries. This type of structure possesses a high aspect ratio, exhibits excellent uniformity at the microscopic scale, thereby enabling the formation of a continuous conductive network and ion transport channels throughout the electrode. Such a unique structural design effectively shortens the diffusion path of lithium ions and alleviates structural fragmentation caused by local stress concentration through axial deformation and radial stress dissipation. By leveraging these characteristics of the 1D structure to optimize the performance of silicon anodes, the superiority of typical structures such as nanotubes and nanowires can be further demonstrated through specific designs.

The hollow structure, as the core feature of the nanotube structure, can effectively mitigate the volume expansion of silicon. Liu et al. designed an NC@Si@CNTs electrode resembling a coaxial cable. Under a current density of 2 A g^−1^, the NC@Si@CNTs electrode maintained a reversible specific capacity of 910 mAh g^−1^ [86]. Liu et al. successfully prepared coaxial silicon-coated carbon nanotubes (CNTs/Si) via a hydrothermal method. During the charge/discharge process, no structural changes were observed in the composite, confirming that the hollow structure design effectively mitigates the drastic volume changes during cycling [87]. Another common structure is nanowires, which can grow directly on the surface of the current collector and ensure efficient electron transport without the need for a large amount of additional conductive agents. Amiinu et al. prepared an electrode composed of SiNWs with indium seeds grown on a 3D Si/C current collector. This binder-free electrode structure enables the formation of a thin active nanowire layer with a thickness of less than 10 μm while achieving a high area loading (1.04 mg cm^−2^) [88].

**Figure 6:**
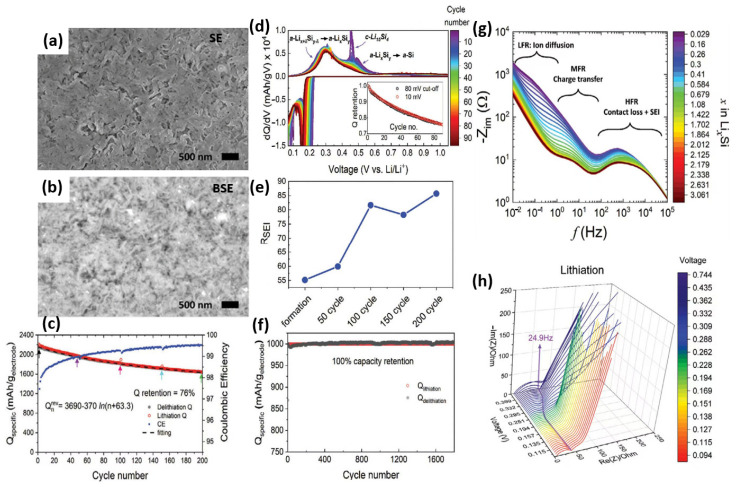
Morphologies and electrochemical performance of 1D structure material (Si NWs network): (**a**) SEM image after 100 cycles. (**b**) Corresponding back-scattered micrograph. (**c**) The specific capacity during cycling at 0.2C across 200 cycles. (**d**) Differential capacity contour plot. The inset: a comparison of capacity retention rates for cells utilizing different cut-off voltages. (**e**) Variation in SEI resistance throughout the cycling process. (**f**) The long cycling performance under a restricted lithiation capacity and at 0.2C rate. (**g**) The imaginary part of in situ impedance (-−Zim) versus frequency in the duration of lithiation process. (**h**) In situ impedance spectra of the anode under varying voltages during the lithiation process. (Reproduced from [89] with permission from Wiley, 2024).

To address the challenges of rapid volume expansion and insufficient conductivity of silicon-based anodes during lithiation/delithiation, which hinder their practical application, the design of hollow structures has emerged as a highly promising strategy in lithium-ion battery research. Pendashteh et al. [89] developed a 100% silicon nanostructure electrode material. The excellent preservation of the network structure highlights the robust mechanical stability of silicon nanowires during repeated volume expansion and contraction (Figure 6a,b). The high-aspect-ratio silicon nanowire network effectively solves the pulverization problem of silicon-based electrodes during cycling by releasing stress. Figure 6c,f shows that the as-prepared material exhibits excellent long-term cycling performance, which is attributed to the nanowire structural design that maintains electrode integrity and prevents pulverization or fracture. After 200 cycle tests, the specific capacity remains at 1640 mAh g^−1^, providing strong evidence of its stability. Figure 6d indicates that, compared with the thin-film electrode, the formation of c-Li_15_Si_4_ phase has a less adverse effect on capacity. During cycling, the SEI resistance of the silicon nanowire electrode exhibited an approximately linear increase with cycle number (Figure 6e). Figure 6g,h indicates that during lithiation, the interfacial resistance and charge transfer resistance of the as-prepared material decreased as the lithiation depth increased. This characteristic reflects excellent interfacial stability and charge transfer kinetics. The nanowire structure effectively promotes uniform SEI formation and optimizes the charge transport pathway.

In research on the structural design of 1D silicon-based anodes, multiple strategies have significantly improved cycling stability and alleviated the volume expansion issue [90,91,92]. These studies indicate that 1D structural design can greatly enhance the electronic transport efficiency and cycling stability of the electrodes, and has become an important approach for balancing performance and practicality. Although 1D structures possess outstanding directional transport advantages, the uniaxial architecture in 1D structures has limited stress dissipation capability in the lateral direction. In contrast, the lateral coating or interlayer confinement of 2D structures can effectively prevent the occurrence of axial fractures.

#### 3.1.3. 2D Structure Design

The 2D structure, with its atomically smooth surface, enables uniform contact between the electrolyte and active material, effectively avoiding the uneven contact caused by the aggregation of 0D particles. Moreover, the adjustable interlayer nanochannels can prevent structural collapse during the volume expansion process. As two typical materials with 2D structure, silicon nanosheets and graphite sheets serve as conductive supports. The performance regulation mechanisms of these two materials are elaborated in the following text.

Silicon nanosheet is a typical representative of 2D structure designs. Nanosheets have precisely controlled thickness and lateral dimension regulation ranging from 200 nm to 2 μm. Their ultrathin thickness and high specific surface area shorten the diffusion path of Li^+^ and enhance the electrode/electrolyte contact. The lithium ion diffusion mechanism is in the form of bulk diffusion in bulk silicon, while it is interlayer diffusion in two-dimensional silicon nanosheets. Interlayer diffusion has a shorter diffusion path compared to bulk diffusion, thereby enhancing the lithium ion transport kinetics. Compared with nanoparticles, nanosheets can provide a more continuous in-plane transport path and better inter-particle connectivity. Qin et al. designed a 2D mesoporous silicon nanosheet/carbon material. Through the structure of nanosheets, the vertical diffusion distance of lithium ions was significantly reduced [93]. Wang et al. prepared a Si nanofilm@rGO composite material on the surface of water-soluble NaCl particles and during the Mg reduction process, where rGO acts as a flexible conductive framework and mechanical supporting skeleton for the electrode [94]. Graphene-based materials represented by a 2D structure exhibit superior mechanical flexibility and high electronic conductivity. Serving as coating layers or supporting skeletons, these materials mitigate the drastic volume variation of silicon, reduce local stress accumulation and restrain electrode cracking. Moreover, they can establish continuous 2D conductive networks within silicon-based electrodes, reduce interfacial contact resistance between particles, and enhance the electronic conductivity. Ma et al. developed a layered Si@graphene structure (p-Si@GN) with improved contact interface through a simple high-pressure method, in which the surfaces of silicon particles were tightly encapsulated by graphene sheets [95]. Furthermore, graphene functions as a buffer skeleton and constructs continuous conductive networks, effectively enhancing the electrochemical performances of the Si anode.

**Figure 7:**
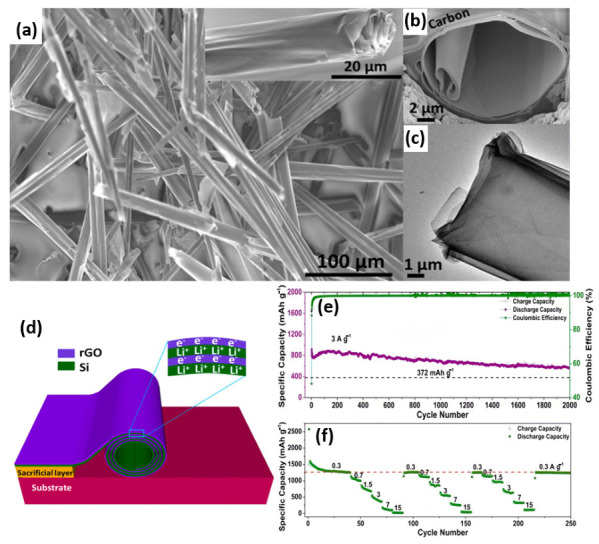
Morphologies and electrochemical performance of 2D structure material (scrolled Si/rGO nanofilms: (**a**) SEM micrograph (inset: an individual nanofilm). (**b**) Cross-sectional micrograph and (**c**) TEM micrograph of an individual nanofilm. (**d**) Illustration of the fabrication procedure. (**e**) Cycling performance at 3 A g^−1^. (**f**) Rate capability within 0.01—1.5 V vs. Li/Li^+^. (Reproduced from [96] with permission from ACS, 2015).

The 2D structure design based on silicon nanosheets and graphene materials improved the performance of the silicon anode by optimizing the lithium-ion transport path and enhancing the structural stability. Liu et al. [96] further adopted the stress-relieving strategy and designed a curled Si/rGO bilayer nanofilm composite material. This double-layer nano-membrane structure with abundant internal voids and multi-layered curled structure is suitable for alleviating the volume expansion of silicon during the cycling process (Figure 7a–d). The internal cavities provide additional buffer space for the volume changes of silicon during the lithiation process. The curled structure achieves stress release through interlayer cooperative deformation, preventing severe cracking of the silicon nanofilm. Moreover, rGO has excellent mechanical flexibility and electronic conductivity, which can maintain the integrity and interlayer contact of the bilayer structure and maintain a stable electronic transmission pathway. Based on the aforementioned structural advantages, the curled Si/rGO double-layer nanofilms can effectively alleviate the degradation of the electrode structure, thereby achieving excellent long-term cycling stability. Figure 7e shows that the sandwich-like structure material exhibits excellent cycling performance under high current density. After 2000 cycles at 3 A g^−1^, the capacity decay is only 3.3% every 100 cycles. As shown in Figure 7f, the material has excellent reversibility. The excellent reversibility stems from the flexible deformation of the silicon nanofilm, which can adapt to volume changes and avoid structural damage.

These studies, along with the relevant supportive findings in reference [75,97], have confirmed the feasibility of the 2D structure design. 2D structure design enables the silicon anode to maintain the advantage of high specific capacity while significantly improving cycling stability and rate performance. The 2D structure enhances the dissipation capacity of lateral stress, but the layered structure exhibits a longer ion transport path in the vertical direction. In contrast, the 3D structure effectively shortens the vertical ion transport distance by constructing interconnected networks.

#### 3.1.4. 3D Structure Design

The 3D structure is regarded as a complex framework, in which the nanoscale basic building units and the 3D interconnected framework are considered as the core design strategies. The nanoscale basic units effectively address the issue of ion transport efficiency, while the 3D interconnected structure effectively tackles challenges related to spatial buffering and structural stability. Unlike the traditional structure that relies on the loose mixture of silicon particles and carbon black conductive agents, this structure integrates silicon nanodomains into the conductive framework, forming a permeable electronic network and significantly enhancing conductivity. Currently, various derivative design strategies, such as the combined design of 3D networks with microsphere structures, as well as fiber network structures, have been extensively explored.

The conductive network with abundant pores and the well-defined microsphere are integrated through interface bonding and spatial encapsulation, effectively addressing the issues of structural collapse and low electrical conductivity. Zhang et al. used SCNTs as the conductive network and Si@C composites as the lithium-ion storage center to construct a scaffold-like network structure [98]. Yi et al. designed an anode structure in which the graphene conductive network completely encapsulates the nitrogen-doped C/Si nanoparticles [99]. The fiber network structure is another common strategy for 3D structures. The core concept of the fiber network lies in fully leveraging the deformability of fibers and the through-hole characteristics of the network to specifically tackle the defects of silicon anodes, specifically, structural collapse and low lithium-ion transport efficiency. Gao et al. fabricated Si/CNTs/CNFs nanocomposites with a standard fiber network structure through electrospinning technology [100]. Ji et al. incorporated silicon nanoparticles into porous N-doped carbon fibers (Si/P-NCFs) via electrospinning and pyrolysis techniques, and these fibers interwove to form a flexible film [101].

**Figure 8:**
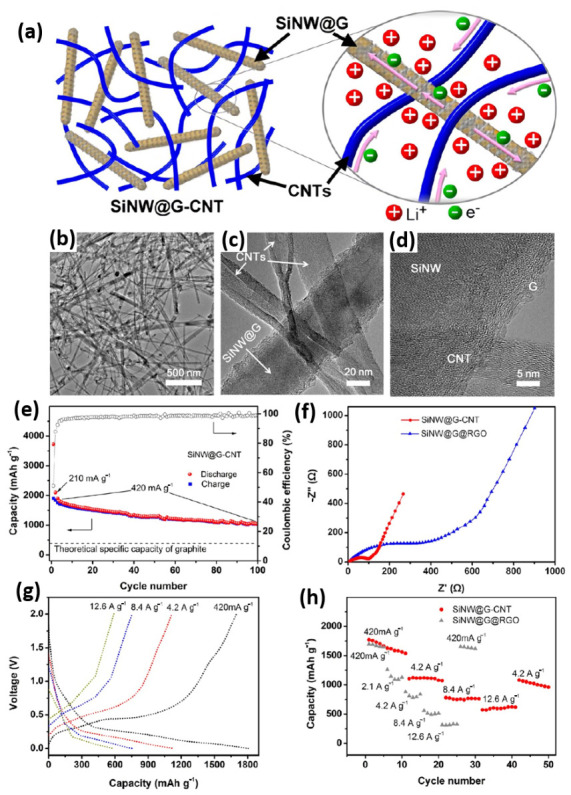
Morphologies and electrochemical performance of 3D structure material (SiNW@G-CNT): (**a**) Schematic diagram depicting the transport of electrons and lithium ions. (**b**,**c**) TEM images. (**d**) High-resolution TEM image. (**e**) Cycling performance. (**f**) EIS profiles. (**g**) Voltage profiles. (**h**) Charge capacities. (Reproduced from [102] with permission from ACS, 2013).

Previous studies focused on 3D structures such as networks, microspheres, and fiber networks, effectively addressing the problems of structural collapse and insufficient electrical conductivity. To further optimize performance, subsequent research shifted toward developing anodes with cross-linked network structures. Wang et al. [102] fabricated a hybrid electrode with a 3D interconnected network structure using Si NWs and CNTs. Figure 8a illustrates the entanglement characteristics of the as-fabricated electrode and the cross-linked network structure that effectively promotes efficient electron and Li^+^ transport during charge/discharge processes. Figure 8b–d illustrates that SiNW@G is uniformly dispersed in CNTs, and the two components are cross-linked to form a 3D network. As shown in Figure 8e, the material exhibits excellent reversible capacity. Figure 8f,h demonstrates that the interconnected porous channels in the 1D composite structure can significantly optimize Li^+^ transport kinetics and reduce the Li^+^ diffusion impedance of the system. Figure 8g proves that the amorphous phase formed during the first charge–discharge process contributes to the excellent high-rate performance.

In the field of silicon-based anode design, many excellent 3D structures have been developed. Chen et al. used a simple and cost-effective magnesium thermal reduction process, with pre-treated sepiolite ore as the raw material to prepare a corn-like structure of silicon (Si) [103]. Men et al. fabricated 3D porous microscale pomegranate-shaped nano silicon/RGO composite material by combining spray drying and magnesiothermic reduction [104]. These studies further demonstrate that the 3D structure design is one of the key directions for achieving practical applications of high-energy-density lithium-ion batteries. In general, these four types of structures have different design focuses. 0D structures emphasize the nanoscale structural units, 1D structures enhance charge transport pathways, 2D structures optimize interfacial uniformity and stress dissipation, while the 3D structures integrate the above advantages and improve the electrochemical performance from multiple aspects. To facilitate comparison of the performance of Si-based anode materials with different dimensional structures, the electrochemical performance of each of the aforementioned types of Si-based anode materials is summarized in Table 2. The 0D and 1D structures exhibit excellent reversible capacity under low current density, but their capacity decays rapidly under high current density or prolonged cycling. The 2D structures exhibit the highest reversible capacities at moderate current densities, while 3D designs excel in long-term cycling performance. Overall, increasing the dimensionality from 0D to 3D enhances structural robustness and electrochemical stability, although sometimes with a trade-off in initial specific capacity.

### 3.2. Architecture Design

The architecture structure design for silicon anodes focuses on core–shell, sandwich-like and network structures. The core–shell structure can effectively alleviate the volume expansion of silicon, the sandwich-like structure can disperse stress through a layered structure, and the network structure builds an efficient electron/ion transmission network.

#### 3.2.1. Core–Shell Structure

Since the pure Si anode undergoes significant volume expansion during the (de)lithiation process and is prone to forming an unstable SEI film at the interface with the electrolyte, a core–shell structure has been adopted in the high energy density LIBs. Currently, this strategy has been widely utilized, as it can protect the core silicon and enhance the electrochemical performance of the electrode. In the core–shell structure, the shell layer is the key factor determining whether the core can achieve high capacity and high stability. A series of studies have optimized the shell structure through various strategies, including interface assembly strategies and interlayer design strategies. Regarding the crucial regulatory role of the shell on the performance of the Si-based anode core, the specific design concepts of two strategies, as well as their specific effects on enhancing electrochemical performance, are elaborated in the following paragraphs.

The interlayer design strategy enhances overall performance by introducing appropriate materials, such as flexible polymers and porous carbon layers, between the core layer and the shell layer. Zhang et al. [105] construct TiSi_2_ layers through high-temperature calcination, thereby preparing Si@TiSi_2_@NC electrodes, which effectively enhanced electron and ion transport. The electrode exhibits an initial reversible capacity of up to 2172.7 mAh g^−1^, and an excellent rate performance of 1198.4 mAh g^−1^ [106]. Xiao et al. prepared a Si@Si_3_N_4_@C electrode with a yolk-shell structure, in which the highly strong and tough Si_3_N_4_ serves as the interlayer material. The embedded capacity of this composite is 91.51%, demonstrating a significant initial discharge capacity of 3093.8 mAh g^−1^. After 200 consecutive cycles, the capacity retention rate exceeds 80%.

**Figure 9:**
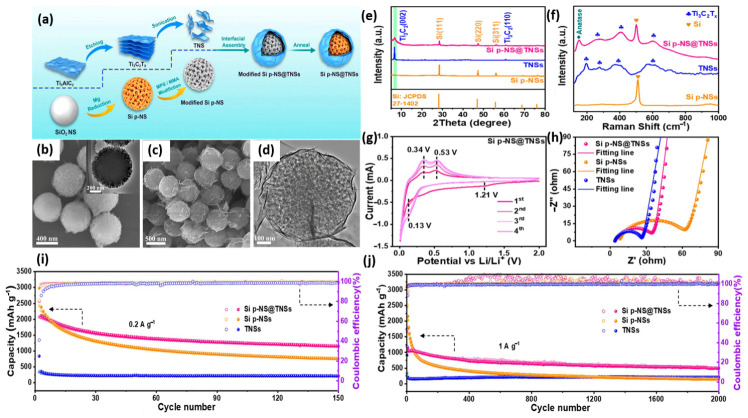
Preparation and electrochemical performance of core–shell structure (Si p-NS@TNSs): (**a**) Preparation process. (**b**,**c**) SEM micrographs: PMMA layer-coated modified Si p-NSs (**b**) and Si p-NS@TNSs composite (**c**). The TEM micrograph of a modifed Si p-NS is the inset in (**b**). (**d**) TEM micrograph. (**e**) XRD pattern. (**f**) Raman spectra. (**g**) CV curves. (**h**) EIS profiles. (**i**) Cycling performance at 0.2 A g^−1^. (**j**) Long cycle performance for 2000 cycles at 1 A g^−1^. (Reproduced from [107] with permission from ACS, 2020).

The interface assembly strategy primarily relies on interfacial tension or chemical bonding to direct the ordered arrangement and immobilization of functional components, such as precursors located at the interface. In this manner, interfacial stability can be optimized and electrode performance can be improved. Xia et al. [107] prepared a composite material with excellent electrochemical performance through using an interfacial assembly strategy. Figure 9a presents a schematic diagram of the synthesis route of Si p-NS@TNSs. Figure 9b–d illustrate the structure characteristics of the coating layer in Si p-NS@TNSs. Figure 9e,f proves that the crystalline Si in the composite is fully integrated with the layered MXene structure without the formation of impurity phases. Figure 9g demonstrates the formation of a stable SEI film on the electrode surface. Additionally, the contact resistance and charge transfer resistance of the composite are much lower than those of pure silicon materials, confirming the marked improvement in conductivity and transport kinetics. This is attributed to the core–shell structure based on the PMMA interface assembly effect, which provides a continuous conductive network for the active Si and strengthens the interface bonding (Figure 9h). Figure 9i,j show that the composite material exhibits excellent long-cycle and high-rate performance.

In a core–shell structure, lithiation of the silicon core induces volume expansion, which can be constrained by the shell, resulting in a stress state dominated by compressive stress. This stress state can be decomposed into radial stress and hoop stress. Radial stress reflects the degree of constraint, while hoop stress is closely associated with crack initiation. In a yolk–shell structure, a pre-designed void space between the silicon core and the shell allows the silicon to expand freely to some extent during the initial stage of lithiation. Once the silicon expands sufficiently to contact the outer shell, it exhibits stress characteristics similar to those of a conventional core–shell structure.

Gao et al. [108] established a stress model for a single core–shell particle under lithiation. In this model, the radial stress $\sigma_{r}$, and hoop stress $\sigma_{\theta }$, in the silicon core are jointly determined by the Si–C interfacial stress and the lithium concentration distribution. The equations are given as follows:

(6) $$\sigma_{r}=\sigma_{r}^{CS}+\frac{2\Omega_{1}E_{1}}{3\left(1-\nu_{1}\right)}\left(\frac{1}{a^{3}}\int_{0}^{a}cr^{2}dr-\frac{1}{r^{3}}\int_{0}^{r}cr^{2}dr\right)$$

(7) $$\sigma_{\theta }=\sigma_{r}^{CS}+\frac{\Omega_{1}E_{1}}{3\left(1-\nu_{1}\right)}\left(\frac{2}{a^{3}}\int_{0}^{a}cr^{2}dr+\frac{1}{r^{3}}\int_{0}^{r}cr^{2}dr-c\right)$$

where $\sigma_{r}^{CS}$ is the radial stress at the Si–C interface, c is the lithium concentration, $\Omega_{1}$ is the partial molar volume of Si, $E_{1}$ is the Young’s modulus of the silicon core, $\nu_{1}$ is the Poisson’s ratio of the silicon core, and a is the radius of the silicon core.

These findings and the corresponding supporting data [76,109,110] confirm that the incorporation of a core–shell structure into the high-energy-density LIBs can protect the Si core and enhance the electrochemical performance of the electrodes. This structural design effectively addresses the severe volume expansion of pure Si anodes during lithiation/delithiation and mitigates the formation of an unstable SEI at the electrode/electrolyte interface.

#### 3.2.2. Sandwich-like Structure

The single-layer core–shell structure has limitations in meeting the long-cycle demands of high-capacity electrodes. In contrast, the sandwich structure overcomes this limitation through a multi-layer design integrating active and functional layers. With this architecture, the middle active layer serves as the core Li^+^ storage region, while the functional layers on both sides play indispensable roles in buffering mechanical stress, improving electrical conductivity, and enhancing interfacial stability. Accordingly, recent research on sandwich structures has mainly focused on the selection of functional- layer materials. MXene and rGO are popular functional layer materials. Additionally, a novel strategy employing continuous fibrous active materials as the functional layers has also been explored. The following paragraphs discuss the working mechanisms and corresponding lithium-storage performance of these two mainstream materials (rGO and MXene), as well as the emerging use of continuous fibrous materials in a sandwich structure electrode.

With abundant modification sites, rGO serves as an effective functional layer material. This structure not only suppresses the active components loss in the intermediate layer but also alleviates volume expansion via its flexible framework. Agyeman et al. [111] prepared a polydopamine-coated Si NPs/rGO composite material and achieved a high reversible capacity of up to 1001 mAh g^−1^, benefiting from its porous structure and the mechanical flexibility of rGO. Based on the 3D network formed by the interweaving of 1D materials, continuous fibrous materials can provide uniformly distributed load-bearing sites and effectively disperse stress, thereby preventing structural cracking. Chen et al. fabricated a sandwich-like anode with a unique nanofiber network through electrospinning. The electrode exhibited excellent Li^+^ storage capability and outstanding cycling stability of 623.4 mAh g^−1^ after 6000 cycles at a current density of 2 A g^−1^ [112].

**Figure 10:**
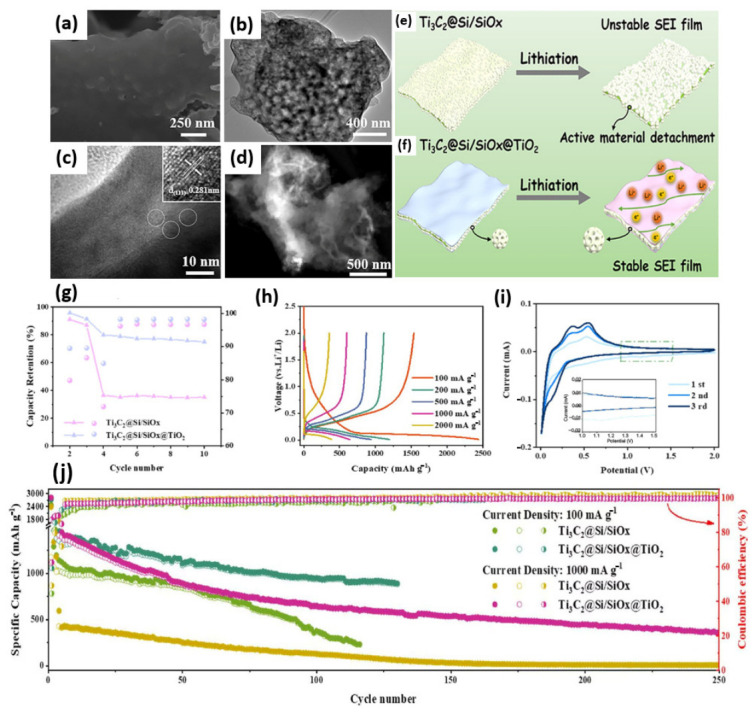
Morphologies and electrochemical performance of sandwich-like structure (Ti_3_C_2_@Si/SiO_x_@TiO_2_): (**a**) SEM image. (**b**) TEM image. (**c**) HRTEM image. (**d**) STEM image. (**e**,**f**) Schematic illustration of the lithiation process. (**g**) Coulombic efficiency and capacity retention at a current density of 1000 mA g^−1^. (**h**) Voltage profiles conducted across a current density range of 100–2000 mA g^−1^ and a voltage window of 0.01–2V. (**i**) CV curves for the first three cycles. (**j**) Cycling performances at various current densities. (Reproduced from [113] with permission from ACS, 2020).

When MXene is used as the functional layer, the performance of the composite can be further enhanced by constructing a coating modified with an amorphous metal oxide. Jiang et al. [113] prepared an interfacial-amorphous composite with a sandwich structure. As shown in Figure 10a–c, the as-prepared composite possesses a smooth surface and is coated with a TiO_2_ layer. The EDS element mapping images clearly demonstrate the uniform distribution of Ti, Si, O, and C throughout the composite (Figure 10d). Figure 10e,f schematically illustrate the lithiation process of the as-prepared composite. Additionally, this anode exhibits stable and high Coulombic efficiency (>98%) (Figure 10h). Figure 10g,i confirm that the amorphous TiO_2_ coating can reduce the consumption of active materials. Figure 10j shows that even after 250 cycles, the anode can still maintain a Coulomb efficiency of 99.6% under a high current density of 1000 mA g^−1^. In the sandwich structure composed of different material layers, the 2D Ti_3_C_2_ forms a laterally continuous conductive network, enabling rapid lateral electron transport and reducing charge transfer resistance. Owing to these structural advantages, the Ti_3_C_2_@Si/SiO_x_ electrode exhibits excellent electrochemical performance.

These studies and corresponding supporting evidence [114,115] confirm that the sandwich structure, through the multi-layer design of the active layer and the functional layer (with the middle active layer serving as the Li^+^ storage center and the two functional layers providing mechanical buffering), not only enhances conductivity but also improves interfacial stability. Therefore, the sandwich structure has emerged as an effective and promising anode design strategy for high-performance LIBs.

#### 3.2.3. Network Structure

The sandwich structure may still suffer from poor structural stability and mass transfer kinetics due to stress concentration and interlayer separation. To address these issues, network structures with 3D interconnected frameworks have been widely introduced into the structural design of high-capacity electrodes. The network structure can not only disperse the volume-expansion-induced stress of active materials in 3D space during the charge/discharge process, but also construct ion/electron transport pathways throughout the material. This design strategy effectively improves the uniform loading of active materials and enhances long-term structural stability. The following paragraphs discuss the structural characteristics and performance-regulation mechanisms of metal-based and carbon-based network architectures.

Metal-based network structures are typically constructed using metals (Ti, Cu, Ni) or metal compounds (TiO_2_, TiN) as the framework. These metallic frameworks provide strong mechanical support and high electrical conductivity, which can suppress the drastic volume changes in Si and establish efficient electron transport pathways. Zhu et al. prepared a Si-Ni nanofoam composite, in which the Ni nanofoam served as the electrode framework, analogous to the steel mesh used in reinforced concrete. The electrode exhibited high areal capacity and excellent cycling stability. After 100 cycles, it delivered an areal capacity of 1.39 mAh cm^−2^, with a capacity retention rate of 69.5% [116]. The carbon-based network structure is composed of carbon materials, such as CNTs and porous carbon as the framework. The intrinsic flexibility of these carbon materials allows them to accommodate the volume expansion of Si during charge/discharge processes, thereby dispersing stress. Guan et al. prepared Cu-coated SiNPs embedded within a 3D CNT network. The composite electrode exhibits excellent rate performance. When the current density was reduced to 100 mA g^−1^, the theoretical specific capacity recovered to 2338.6 mAh g^−1^, while maintaining a capacity retention of nearly 80% [117].

**Figure 11:**
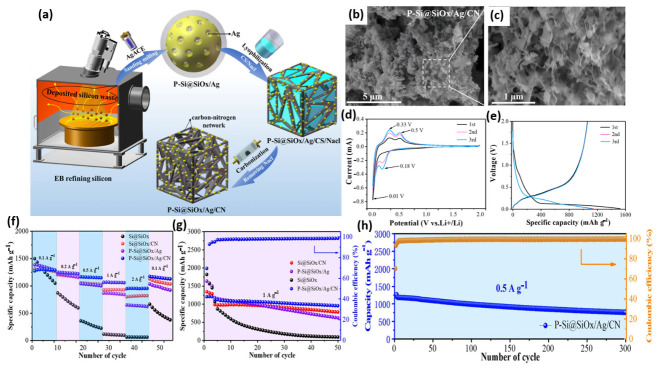
Preparation and electrochemical performance of network structure (P-Si@SiO_x_/Ag/CN): (**a**) Schematic illustration of the synthesis process. (**b**,**c**) SEM micrographs. (**d**) CV curves and (**e**) galvanostatic lithiation/delithiation profiles for the first three cycles at 0.2 A g^−1^. (**f**) Rate performances. (**g**) Cycling performance at 1 A g^−1^. (**h**) Long cycle performance at 0.5 A g^−1^. (Reproduced from [118] with permission from ACS, 2023).

Doped carbon materials with abundant pores represent another important class of carbon-based network materials. When combined with silicon, the pore spaces within these carbon materials can help alleviate the volume expansion of silicon during cycling. Heteroatom doping in the carbon framework can further improve the conductivity. Li et al. [118] used a C-N grid with 3D interconnection and porous structure as the substrate to synthesize the P-Si@SiO_x_/Ag/CN electrode. Figure 11a presents a schematic diagram illustrating the synthesis route of the P-Si@SiO_x_/Ag/CN composite. The unique 3D interconnected structure provides significant buffer space, effectively alleviating the volume expansion of Si during the lithiation process (Figure 11b,c). The CV curves of the composite exhibit regular peak shapes and a low polarization, indicating excellent electrochemical reversibility and structural stability during subsequent cycles (Figure 11d). The ICE of the as-prepared material is 69.6% (Figure 11e). Figure 11f demonstrates the excellent reversibility of the material. This behavior is attributed to the three-dimensional network structure of carbon-nitrogen materials, which acts as a rigid framework to maintain structural integrity and provide continuous conductive pathways as well as rapid ion transport channels. Figure 11g,h indicates that the composite electrode has excellent capacity retention performance.

These studies and the corresponding supporting evidence [119,120,121] demonstrate that network structures can effectively disperse the volume-expansion-induced stress of active materials during the charge/discharge process while simultaneously constructing ion/electron transport pathways throughout the material. As a result, network structures significantly improve the uniform distribution of active materials and enhance long-term structural stability. Therefore, the network structures have been widely applied as an effective strategy for improving the practical performance of LIBs. In order to facilitate the comparison among Si-based anode materials with different architecture designs, the electrochemical performance of the various types of Si-based anode discussed above is summarized in Table 3. Core–shell architectures generally provide high reversible capacity and excellent long-term cycling stability because they effectively buffer volume expansion. Sandwich-like structures exhibit moderate electrochemical performance with relatively stable capacity retention. Network structures, often incorporating carbon nanotubes or conductive frameworks, typically achieve the highest initial capacities, although they are prone to experiencing faster degradation during prolonged cycling. Overall, core–shell and network designs demonstrate superior rate capability and capacity retention, highlighting the importance of architectural engineering in enhancing the electrochemical stability and structural integrity of Si-based anodes for LIBs.

## 4. Future Challenges

Although silicon has attracted attention due to its excellent theoretical specific capacity, it still faces significant challenges in achieving commercialization. The main challenges lie in the mechanism analysis of silicon during the de(lithiation) process, the customized design of the structure, the assessment of costs, safety and scalability in achieving industrial mass production, as well as the improvement of the full-cell performance.

### 4.1. Mechanism Elucidation

Although silicon-based anodes have been widely used in LIBs, the related mechanisms, including the electrochemical mechanisms of alloying/dealloying reactions, the mechanisms underlying structural evolution and stress–strain, the formation and evolution mechanisms of the SEI film, and the analysis of failure mechanisms, have not been clearly elucidated. This seriously hinders the commercialization of silicon anodes. In situ characterization techniques can capture the reaction process of the electrode in real time, monitor the dynamic changes in the composition of the SEI film, analyze the phase transition and Li^+^ diffusion mechanism of the electrode during de(lithiation) process, and thereby effectively refine theoretical models and guide battery optimization.

#### 4.1.1. Function of Individual In Situ Characterization

Although scholars from various countries have utilized a wide range of in situ characterization techniques to reveal many new reaction mechanisms of silicon-based anodes under specific conditions, the current in situ characterization techniques still face many challenges, including damage to the sample structure, limitations in spatial resolution and limited cycle times. Therefore, continuous efforts are still needed to further elucidate the mechanisms of silicon-based anodes.

**Figure 12:**
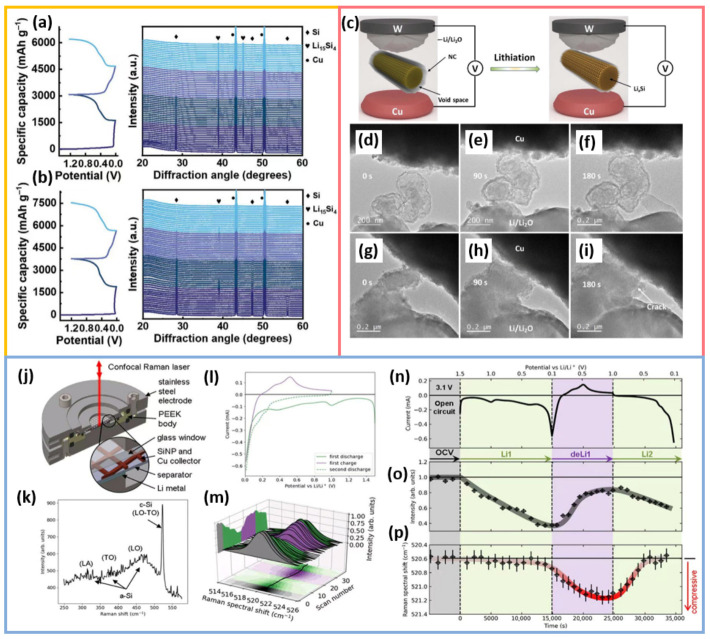
In situ characterization of Si-based anode materials: in situ XRD profiles of pristine Si (**a**) and Si@SiOC electrodes (**b**) over the first two charge–discharge cycles. (Reproduced from [127] with permission from Wiley, 2024). (**c**–**i**) p-Si NRs@void@NC: (**c**) Electrochemical cell schematic for TEM interior. Dynamic time-lapse visuals of (**d**–**f**) p-Si NRs@void@NC and (**g**–**i**) p-Si NRs@NC during lithiation. (Reproduced from [132] with permission from ACS, 2017). (**j**–**p**) SiNPs: (**j**) Electrochemical cell and experimental in situ Raman configuration. (**k**) Typical Raman spectra. (**l**) Cyclic linear sweep voltammograms. (**m**) TO-LO peak variation in c-Si with scan number across open-circuit (OCV), discharge (green) and charge (purple) cycles. (**n**) Current, (**o**) Raman peak intensity, and (**p**) spectral position variation with time and voltage. (Reproduced from [137] with permission from ACS, 2017).

In situ XRD can detect phase transitions within the 0.1% range and quantify the rate of capacity decay of the electrode [124,125,126]. Hernandha et al. [127] used in situ XRD to track the formation/elimination process of the Li_15_Si_4_ phase in real time, as well as to monitor the crystal orientation and size of Li_15_Si_4_, thereby providing a clear explanation of the crystal phase evolution mechanism (Figure 12a,b). In situ TEM, based on the real-time detection of morphological features at the nanometer/atomic scale, can effectively clarify issues related to the microscopic failure mechanism of the electrode [128,129,130,131]. Du et al. [132] used in situ TEM to reveal the lithiation mechanism of the p-Si NRs@void@NC composite electrode. Figure 12c–i show that during the lithiation process, Li^+^ passes through the NC shell and forms alloys, causing the p-Si NRs to expand in volume and almost fill the entire cavity. In situ Raman, by tracking the stress changes in the crystal during the de(lithiation) process, provides molecular-level evidence for the battery reaction mechanism and degradation mechanism [133,134,135,136]. Tardif et al. [137] used in situ Raman to clarify that the capacity decay mechanism of the electrode under long-term cycling is related to the continuous accumulation of compressive stress (Figure 12j–p). To enable a more systematic and multidimensional comparison from multiple perspectives, this review summarizes various in situ characterization techniques as shown in Table 4. This table provides guidance about characterization techniques for studying the dynamic evolution of the SEI and structure of silicon anodes, and assesses different characterization techniques from the perspective of practical operability. However, due to limitations such as spatial/time resolution and detection sensitivity, individual in situ characterization still has its shortcomings. Therefore, in recent years, a large number of studies have further improved the accuracy of mechanism elucidation for silicon-based anodes by coupling various in situ characterizations.

#### 4.1.2. Coupling of Various In Situ Characterization

In order to fully reveal the actual changes in the material, a large number of studies have employed the combined application of different in situ characterization techniques. Zhang et al. used the combination of in situ XPS and in situ XRD to reveal the source of the thermal stability advantage of the NCM-SC cathode in LIBs, including the single crystal structure that inhibits lattice distortion and the formation of a stable SEI film on the surface to reduce thermal decomposition, providing key insights for mitigating the thermal runaway of full-cells [138].

Variations in silicon particle size distribution, nonuniform carbon-coating thickness, and differences in the porosity can lead to deviations in mechanistic interpretation when different in situ characterization techniques are used for analysis. However, the strategy of fabricating dual windows on a single in situ cell can eliminate these deviations. In studies revealing the reaction mechanisms of Si-based anodes in LIBs, integrating two windows on a single in situ cell and conducting two types of in situ characterizations simultaneously can enable the analysis of the same reaction region under identical electrochemical operating conditions. This is an effective strategy to enhance the accuracy of mechanism analysis. Vanpeene et al. conducted in situ XRCT and in situ XRD simultaneously on the same in situ cell, revealing the core mechanism of capacity fading. In situ XRCT observed the dynamic changes in the electrode structure, including electrode expansion/contraction and porosity evolution. In situ XRD analyzed the crystal structure and phase transformations. In situ XRCT and in situ XRD together proved that the capacity fading was caused by porosity reduction due to SEI accumulation rather than the formation of harmful phases, providing valuable insights for addressing the capacity fading issue [139].

Although in situ characterization has demonstrated significant advantages in revealing the mechanism, it still faces several challenges, such as the limitation of only being able to test a limited number of cycles, the unclear mechanisms and changes during the continuous cracking and regeneration of SEI, and the difficulty in tracing the gradual collapse of the conductive network. In addition, the sensitivity threshold remains one of the core bottlenecks of in situ characterization. The intermediate reaction products with low content and short lifetimes cannot be detected, resulting in unclear elaboration of the reaction pathway and incomplete analysis of the failure mechanism. Therefore, developing long-cycle in situ characterization, combining it with ex situ characterization at specific cycle numbers, and integrating different in situ characterization techniques are key development pathways for achieving the next-generation efficient in situ characterization.

### 4.2. Structural Customization

With the rapid development of new energy vehicles, portable electronics and energy storage devices, the application scenarios of LIBs have exhibited a diversified trend, significantly stimulating the demand for excellent performance. High energy, power, and temperature adaptability, as two major challenges in current diversified application scenarios at present, have imposed specific requirements on the structural design of Si-based anodes in the LIBs. Since conventional structural design struggles to meet the performance demands under diversified scenarios; therefore, a customized design of the structure of Si-based electrodes is necessary.

**Fast charging:** The demand for fast charging in the new energy field is driving the development of Si-based anodes towards high-rate performance. However, in practical applications, silicon undergoes intense volume changes, leading to the irreversible collapse of the conductive network under high current density. The pulverization of silicon causes it to detach from the conductive network and become inactive “dead silicon”, resulting in irreversible capacity degradation. In addition, severe concentration polarization and electrochemical polarization occur during large-current charge/discharge, causing the precipitation of metallic lithium. Furthermore, the repeated rupture and regeneration of the SEI film increase interfacial impedance, while difficulties in achieving high areal capacity and high compaction density result in a lack of engineering feasibility.

**Low temperatures:** At low temperatures, Li^+^ diffusion through the SEI is severely hindered, while charge transfer resistance and interfacial impedance increase significantly. Due to the remarkably reduced solid-phase diffusion coefficient, silicon materials are prone to develop concentration gradients, characterized by preferential lithiation at the surface and delayed lithiation in the interior. This nonuniform lithiation behavior leads to stress concentration and may ultimately induce particle cracking. Low temperatures also pose substantial challenges for the electrolyte. The electrolyte systems suitable for room temperature often fail to form an ideal SEI film at low temperatures. Traditional electrolytes with a high proportion of organic components tend to lead to reduced interfacial mechanical robustness. Changes in the electrolyte decomposition path result in increased side reactions. Moreover, the reduced mechanical compatibility between the binder and the electrode as a whole cannot be ignored. Polymeric binders are prone to exhibiting reduced toughness at low temperatures and an increased binding strength with the silicon.

**Wide temperature range:** The wide temperature range performance requires that the silicon anode maintains a high Coulomb efficiency, stable cycling performance, and a controlled volume expansion rate within a wide temperature range. Achieving such performance depends on maintaining multi-scale stability under coupled temperature effects. However, the dominant failure mechanisms vary in different temperature ranges. At low temperatures, interfacial impedance significantly increases. At room temperatures, volume expansion becomes the main cause of capacity degradation. While at high temperatures, side reactions are aggravated. As a result, an optimization strategy modified for one temperature range may lead to deterioration of material performance in another temperature range. Moreover, the temperature adaptability of the SEI is central to wide temperature range performance. A temperature-adaptive SEI design relies on a comprehensive understanding of the SEI dissolution and re-deposition behavior, the balance of elasticity and rigidity, thermal stability, and the evolution laws of these aspects with temperature changes. Additionally, the components of the silicon anode are very complicated. The thermal expansion coefficients, interfacial adhesion, and chemical stability of different components vary at different temperatures. Therefore, the wide temperature range design for the silicon anode requires coupled analysis of the physical and chemical fields of electrochemistry, thermal effects, and mechanical behavior in order to clearly elaborate on the design strategies of silicon-based anode structures in different application scenarios. From the perspectives of failure mechanisms and key performance requirements, this review summarizes and analyses the relevant design considerations, as shown in Table 5. It demonstrates that the failure mechanisms of silicon-based anodes vary in three scenarios. Therefore, customized structural designs are required. The structure in the fast-charging scenario needs to balance rapid kinetics and structural stability. The structure for the low-temperature scenario should reduce transport resistance and stabilize the interface. The structure for full temperature range applications should achieve a controllable volume expansion rate and compatibility across temperature ranges. Overall, each scenario demands tailored structural designs to address specific failure mechanisms while meeting key performance requirements.

### 4.3. Cost, Scalability and Safety

Silicon is abundant in supply and low in cost. However, strategies to mitigate the degradation mechanisms of silicon, such as structural design and pre-lithiation, significantly increase processing costs. Over the past decade, the structural engineering of silicon anodes has evolved from directly using micrometer-sized silicon to nanoscale structures, core–shell structures, and a series of complex designs. These intricate structural designs effectively alleviate volume expansion and stabilize the SEI. However, the increased number of processing steps and specialized equipment raises the material loss rate, prolongs the fabrication process, and adds equipment renovation costs. Moreover, nanoscale structures significantly intensify interfacial side reactions, leading to increased electrolyte consumption and reliance on additives. Pre-lithiation, due to the addition of extra independent processes, strict environmental condition requirements, high demands for process windows and uniformity, introduces additional manufacturing costs. Environmental protection and green production costs are often underestimated issues in the industrialization of silicon anodes. Relevant expenses, such as those for waste liquid and waste residue treatment, carbon emission constraints for high-energy-consumption processes, and volatile organic compound (VOC) control costs associated with organic solvent systems, will gradually increase as environmental protection policies become more stringent.

Scalability remains the main challenge currently faced by silicon anodes. The complex structure design depends on precise control of parameters such as shell thickness, cavity size, and pore size distribution. However, in large-scale production, there are substantial batch-to-batch variations in particle size distribution, difficulty in maintaining the uniformity of carbon coating thickness, and deviations of pore diameters from the designed values. Industrialization requires that silicon electrodes operate stably under high areal capacity conditions and in full-cells with limited electrolyte. However, silicon anodes are prone to local over-lithiation, stress accumulation, and structural degradation under high loading. Additionally, the content of electrolyte in full-cells is strictly limited, making it difficult to meet the need for silicon anodes to continuously consume electrolyte to repair the SEI during the cycling process. Large fluctuations in raw material quality also significantly reduce the scalability. Silicon materials from different processes (mechanical ball milling, chemical synthesis, vapor deposition) differ substantially. Moreover, different enterprises have inconsistent demands regarding particle size, morphology, and surface oxide layer.

The unstable SEI is one of the typical safety risks associated with silicon anodes. The cracked SEI leads to the exposure of active silicon and triggers the decomposition of the electrolyte, thereby intensifying side reactions and increasing the release of gas, which results in an expansion of the battery cell. The expanded battery cell further aggravates the deterioration of the interface contact state and induces localized heat accumulation. The degradation of electrode structure is also a safety issue that cannot be ignored, as it can easily cause an abnormal local potential distribution. The risk of lithium plating is significantly increased under fast charging and low temperatures. The growth and deposition of lithium dendrites in the voids intensify the internal stress, thereby damaging the electrode structure. To improve the initial Coulomb efficiency and the energy density, the pre-lithiation strategy is widely used. However, the materials containing active lithium used in the pre-lithiation process are highly sensitive to water and oxygen, and are prone to releasing heat upon contact with water and undergoing spontaneous oxidation upon exposure to oxygen. In addition, uneven pre-lithiation causes local lithium concentrations to be excessively high and chemical properties to be too strong in certain areas. These areas are more likely to undergo intense side reactions with the electrolyte. In order to effectively address the problem that some silicon anodes cannot achieve commercial application, this review compares the preparation methods of different silicon materials in terms of preparation cost, safety and scalability, as shown in Table 6. Mechanical milling and spray drying offer low to medium cost, medium safety, and high scalability, making them suitable for industrial production. Chemical vapor deposition, template, hydrothermal, and sol–gel methods generally involve higher costs and lower safety due to toxic precursors, high-pressure conditions, or flammable solvents. Their scalability ranges from moderate to low, primarily limited by batch processing, uneven reaction conditions, or complex post-treatment steps. Overall, simple physical methods favor large-scale production, while chemical methods provide greater structural control at the expense of practicality.

From a commercialization perspective, micro-scale Si/C and SiO_x_/C composite materials prepared by scalable methods such as mechanical ball milling and spray drying are considered to have a higher degree of marketability. The SiO_x_/C material developed by Shin-Estu Chemical Co., Ltd. (Tokyo, Japan) and BASF Shanshan Battery Materials Co., Ltd. (Changsha, China) has been widely applied in high-energy-density lithium-ion batteries. Group 14 Technologies (Woodinville, WA, USA), Sila Nanotechnologies (Alameda, CA, USA) and OneD Battery Science (Moses Lake, WA, USA) are currently vigorously promoting a new generation of highly scalable micro-scale Si/C materials. Micro-scale Si/C and SiO_x_/C composite materials have a moderate specific capacity, but their compaction density and production cost are more in line with the requirements of practical production applications. By contrast, the yolk-shell structure, hollow structure, and template-derived porous silicon structure exhibit excellent rate capability, but the high cost resulting from the complex preparation process, low compaction density and high electrolyte consumption significantly reduce their market potential. The current best practice for silicon-carbon anodes is the CVD method. This approach does not require pre-lithiation treatment, allowing for precise control of the silicon particle size within 10 nm. The fabricated electrodes have controllable expansion rates and exhibit the most comprehensive performance that is closest to the requirements for large-scale commercial use. Moreover, the materials obtained through the CVD method can be directly used in the existing lithium-ion battery electrode manufacturing lines, without the need for large-scale line modifications. Tests conducted under conditions of low active material loading and excessive electrolyte, ignoring compaction density and electrode expansion, and neglecting safety and environmental costs during the pre-lithiation process, are common mistakes in the preparation route of silicon anodes for industrialization. Moreover, there are often cases where electrode electrochemical performance tests are conducted under non-standardized or non-practical industrial application conditions. Additionally, some works only focus on the best laboratory data, ignoring the reproducibility of the results. Cost analysis is often based on assumptions and ignores factors such as process complexity, scalability, and manufacturing losses. Therefore, when evaluating materials or structures that are close to commercialization, attention should be paid to tests conducted under standard conditions, data with statistical significance, and work with high scalability in the preparation process.

### 4.4. Full-Cells

Due to the increasing commercial demand for LIBs, the practical application performance of silicon anodes needs to be improved. To evaluate the practical applicability of silicon-based anodes, the electrochemical performance of full-cells assembled with silicon-based anodes and various cathodes is summarized. The relevant results are presented in Table 7. The counter electrode (CE) of the half-cell is a metal lithium sheet, which has the ability to compensate for unlimited lithium content, thus making it impossible to determine the matching situation between the anode and cathode, and evaluate the actual performance of the silicon anode. The full-cell, composed of the cathode and the electrolyte with limited lithium content, can more comprehensively reflect the actual application performance of the silicon anode. This difference in lithium inventory leads to distinct degradation mechanisms between half-cells and full-cells. During the initial lithiation, a large amount of lithium source is used to form the SEI film, and the repeated regeneration of the SEI film during the cycling process continuously consumes the limited lithium source of the full-cell, resulting in insufficient lithium content and a significant decrease in the capacity. In addition to the aforementioned failure mechanism, the low polarization phenomenon in the full-cell poses a high areal capacity demand for the silicon anode. However, the high areal capacity silicon electrode has a significant Li^+^ concentration gradient, uneven local lithiation leading to stress concentration, and a decrease in the utilization rate of the active material with the increase in thickness. Therefore, alleviating the failure mechanism in the process of transforming high specific capacity silicon anodes into high-areal-capacity silicon anodes is a major challenge for improving the performance of the full-cell.

In addition to electrochemical performance, safety is also a key criterion for evaluating the practical applicability of a full-cell. Representative safety tests for full-cells include the nail penetration test, overcharge test, and thermal ramp test. The nail penetration test is used to simulate severe internal short circuits caused by mechanical damage. In silicon-based electrodes, inhomogeneous lithiation and structure degradation are prone to causing internal short circuits. The overcharge test charges the battery to the rated cut-off voltage or above the rated capacity, thereby testing the ability to resist exothermic secondary reactions and lithium desorption. Excessive irreversible lithium consumption and low interface stability are prone to cause overcharging. In the thermal ramp test, the battery is heated at a fixed heating rate continuously to analyze the interface decomposition of the electrode under high-temperature conditions, material heat release, and thermal runaway trigger characteristics. These tests indicate that optimization should be carried out in terms of material structure design, interface design, and battery design to effectively reduce the safety risks of full-cells. In terms of material structure, designing nanostructures, porous hollow structures, and applying high mechanical stability conductive frameworks can effectively alleviate stress concentration and inhibit electrode cracking. In terms of the interface, improving the mechanical and thermal stability of the interface film and reducing the reactivity of the lithiated silicon surface can inhibit the repeated cracking and regeneration of the SEI and lithium loss. In terms of battery design, further optimizing the N/P ratio and reasonably designing the electrode surface loading are also necessary. In addition, using heat-resistant separators, flame-retardant electrolytes, and efficient thermal management systems is also a very important safety protection strategy.

## 5. Conclusions and Perspective

This review comprehensively explores various Si-based anode materials, the structural design of Si-based anodes and their electrochemical properties in LIBs, and thoroughly discusses the challenges and feasible optimization strategies for their practical application. As representative examples of distinctive Si-based anode materials, the different lithium storage mechanisms and targeted design strategies of silicon oxides, silicon nitride, and silicon phosphide are analyzed in detail. Through dimensional design (0D, 1D, 2D and 3D) and architecture design (core–shell, sandwich-like and network structure), the various structural design of Si-based anodes not only provides buffer space for the huge volume expansion during the lithiation process, but also alleviates the problems of electrode cracking and pulverization and other structural failures. In addition, the challenges faced by Si-based anodes in future development, including the limitations of in situ characterization techniques, customized structures under the demand for high-energy-density LIBs and extreme temperatures, commercialization, and the performance of the full-cell, are comprehensively discussed. Although significant progress has been made in the structural design of silicon anodes, the increasing demand for high-energy LIBs and the need for commercialization require continuous optimization through multiple constructive research directions to specifically address the current challenges faced by Si-based anodes.

(1) The sophisticated structural design of Si-based anodes is crucial for addressing the inherent problems of these materials, including severe volume expansion and low electrical conductivity, and for facilitating industrialization. Advanced technologies, such as 3D printing, overcome the limitations of traditional processes by enabling precise control over molding parameters and structural models. 3D printing enables the fabrication of customized Si-based anode structures and the construction of 3D continuous conductive networks, thereby promoting the synergistic transport of electrons and ions.
(2) In the traditional manufacturing process of Si-based anodes, the lack of precise control over process parameters often leads to material defects, such as excessive impurities, coating fractures, and the uneven distribution of structural pores. Big data analysis and machine learning can optimize process parameters to precisely control the composition and structural configuration, thereby overcoming these limitations. Neural networks and random forest models can effectively reveal the implicit relationship between processing conditions and material performance. Moreover, multi-objective optimization algorithms like NSGA-II can achieve precise matching between process parameters and structural design specifications.
(3) The Si-based anode has an extremely high theoretical capacity (4200 mAh g^−1^), significantly higher than that of graphite. However, there is still a considerable gap between the practical performance and the theoretical value of the Si-based anode. To bridge this gap, several aspects need to be improved to enhance the practical performance. Firstly, using in situ XRD and DFT calculations to clarify the phase transition path during lithiation and to design structures with low diffusion barriers to address the kinetic limitation issue. Secondly, self-repairing systems with dynamic covalent bonds to alleviate structural fatigue caused by cyclic stress. Thirdly, effective matching of high-capacity Si-based anodes with cathodes and electrolytes in the full-cell should be achieved to prevent performance degradation due to insufficient lithium supply.
(4) The lithium storage process of Si-based anodes is rather complex, and traditional characterization methods are unable to achieve real-time correlation between structure and performance. Next-generation in situ characterization techniques can precisely identify the key rate-limiting factors during the lithium storage process. This can be achieved by simultaneously acquiring and separating signals from the electric field, stress field, and concentration field. Moreover, through real-time detection of dynamic interface changes, an intrinsic correlation between dynamic interface stability and cycle life is accurately established. The future development of in situ characterization techniques requires improved spatial and temporal resolution to track transient processes and the use of machine learning for advanced multimodal data fusion to reduce signal interference from components like electrolytes, thereby enhancing the accuracy of mechanism analysis.
(5) Experimental studies have limitations in describing the microscopic mechanism of lithium storage. Atomic-scale simulations and electronic structure calculations can effectively reveal the fundamental lithium-storage processes. They can also accurately describe interfacial electronic states and reaction energy barriers. Although progress has been made, there are still challenges in model mismatch and multi-scale coupling. Therefore, it is essential to further develop multi-scale computational methods. It is also important to combine them with machine learning for parameter calibration. These efforts can improve model accuracy and bridge the gap between theoretical and experimental results.

## Figures and Tables

**Table 1:** Electrochemical performance and volumetric stability of diverse Si-based anode materials.

Si-Based Anode Materials	Samples	Volume Expansion	Electrode Loading (mg cm^−2^)	Areal Capacity (mAh cm^−2^)	Current Density (A g^−1^)	ICE	Cycle Number	Reversible Capacity (mAh g^−1^)	Ref.
Silicon	Si@C-650	280−320%	1.0–1.2	0.87	1	84%	200	198.1	[75]
	YS-Si@NC-60	280−320%	—	7.429	0.1	82.2%	110	1446	[76]
Silicon oxide	SiO@C	200−260%	10	1.52	0.152	66%	50	1490	[63]
	SiO@ZnO	200−260%	2.6–4	1.27	0.1	81.2%	500	978.65	[65]
	SiO_x_/C-CVD	220−250%	1.5–2.0	1.75–2.33	0.1	67.4%	150	981	[66]
Silicon nitride	Si_3_N_4_	200−240%	—	—	0.085	—	150	400	[67]
	SiN_0.92_	210−250%	—	—	0.18	Over 80%	100	1300	[68]
	SiN_0.7_	220–260%	0.9–1.1	1.23–1.50	0.684	54.3%	300	≈1123	[77]
Silicon phosphide	SiP	280−320%	—	—	0.1	32%	50	550	[71]
	SiP_2_/nanocarbon	300−340%	1.5	2.37	0.1	88%	100	1515	[73]
	MWCNT-coated SiP_2_	300–340%	1.5	1.52	0.5	73%	100	1622	[74]
	SiP_2_	300–340%	1.5	1.47	0.149	84%	30	980	[78]

**Table 2:** Electrochemical performance of Si-based anode materials with different dimensional structures for LIBs.

Dimensionality	Materials	Electrode Loading (mg cm^−2^)	Areal Capacity (mAh cm^−2^)	Current Density (A g^−1^)	ICE	Cycle Number	Reversible Capacity (mAh g^−1^)	Reference
0D	PoHC@Si@C	2.5	3.53	0.3	83.2%	80	857	[79]
				1.5		200	550	
	p-Si@DCN	—	—	0.1	78%	50	1710	[83]
				1		300	1161	
1D	NC@Si@CNTs	—	—	0.2	62.5%	150	1752	[86]
				2		150	910	
	CNTs/Si/C nanotubes	—	—	0.5	66.2%	1000	1508.5	[87]
				2		1000	932.2	
2D	pSi@C	0.39–0.52	0.872–1.163	1	—	150	2236	[93]
				5		500	467.8	
	GS@Si@C	1	1.48	0.2	84%	100	1471.81	[97]
				2		600	1028.5	
3D	N-C/Si@G	—	—	0.5	≈86%	500	1190	[99]
				5		800	701.4	
	N, P doped Si/CNTs/CNFs	—	—	0.2	≈67%	100	1142	[100]
				2		500	401	
	Si/P-NCFs	0.5	0.693	0.1	67.24%	100	1386	[101]
				1		1000	942	
	Si with a corn-like structure	0.71–0.80	0.56–0.63	0.2	75.1%	200	1447	[103]
				1		500	788	

**Table 3:** Electrochemical performance of Si-based anode materials with different architectures in LIBs.

Structure	Materials	Electrode Loading (mg cm^−2^)	Areal Capacity (mAh cm^−2^)	Current Density (A g^−1^)	ICE	Cycle Number	Reversible Capacity (mAh g^−1^)	Reference
Core–shell structure	Si@TiSi_2_@NC	0.7	1.52	0.2	83.6%	100	1553.3	[105]
				2		1000	847	
	HD–Si@C	1.19–1.33	0.85–0.95	0.2	54%	250	927.1	[109]
				5		3000	713	
	Si-PEI@ZIF-67	—	—	1	65.8%	500	1134.4	[110]
				5		200	828.7	
Sandwich-like structure	Ti_3_C_2_@Si/SiO_x_@TiO_2_	0.8–1.1	0.75–1.03	0.5	66.3%	100	720	[112]
				1		250	365	
	B-Si@SiO_x_/C-700	1.0	0.66	1	63.1%	500	659.3	[114]
				2		1000	460	
Network structure	Si@Cu/CNTs	—	—	0.1	82.68%	50	2107.5	[117]
				1		100	1676.1	
	Si@Fe_3_C@PC	—	—	0.1	62.6%	380	1320.1	[121]
				3		1500	680	
	3D porous Ni/Si	0.3	0.73	0.84	60%	100	2025	[122]
				4.2		100	1420	
	Si/Ni/C	1.4	1.46	0.2	77.3%	100	1044	[123]
				2		500	511	

**Table 4:** The comparison of various in situ characterization techniques.

In Situ Characterization Techniques	Principle	Key Function	Advantages	Disadvantages	Spatial Resolution
In situ TEM	The transmission electron beam penetrates the sample, interacts with the atoms and generates signals.	1. Evolution of electrode morphology 2. Nucleation, growth and reconfiguration process of SEI film.	High spatial resolution, capable of reaching the atomic level.	Requires ultra-thin samples (≤100 nm).	0.1 nm
In situ SEM	The electron beam scans the surface of the sample, exciting secondary electrons/backscattered electrons.	1. Evolution of electrode morphology 2. Detection of the growth state of lithium dendrites.	The field of view is wide, and the characterization results are close to the overall characteristics of the sample.	The measurement accuracy is not as high as that of TEM.	μm~nm
In situ XRD	X-rays irradiate the sample, interacting with the crystal lattice to produce coherent diffraction.	1. Crystal structure and phase transition 2. Quantitative analysis of the variation in phase content with potential.	In situ XRD can detect the overall structure and composition of the sample.	Generally, it can only analyze crystalline substances.	μm~nm
In situ Raman	The sample is exposed to laser light, which excites the molecular/atomic vibrations and generates Raman scattering light.	Identify the formation of crystalline/non-crystalline Si and Li_x_Si alloys.	Strong ability to analyze small areas.	1. The spatial resolution is limited by the laser spot. 2. It is susceptible to fluorescence interference.	10 nm~1 μm
In situ EIS	Apply a small sinusoidal voltage/current, measure the impedance response at different frequencies.	Analyze the impedance of the SEI film, as well as the changes in charge transfer resistance and bulk Li^+^ diffusion resistance.	1. Sensitive to interface dynamic changes. 2. Without sample damage, it can achieve long-term monitoring.	No spatial resolution, only the average resistance of the entire battery can be obtained.	−
In situ XPS	X-rays irradiate the sample, exciting the photoelectrons. The surface elements can be determined by analyzing the binding energy/peak shape of the photoelectrons.	1. Analyze the chemical composition of the SEI film. 2. Real-time monitoring of the growth process of the SEI film.	The detection sensitivity for thin surface films (at the nanometer level) is extremely high.	It can only analyze the surface information of the sample.	≈μm
In situ XRCT	X-rays penetrate the sample. Different phases within the sample absorb and scatter X-rays differently; hence, a three-dimensional structure image of the sample is obtained.	1. Non-destructive monitoring. 2. Monitoring the three-dimensional growth path of lithium dendrites.	It enables three-dimensional morphology analysis.	The test takes a long time and has a low time resolution.	≈μm
In situ AFM	Through the tiny displacement of the probe, changes in the force on the surface are sensed and converted into surface topography images.	Monitor the morphological evolution and the mechanical properties of the electrode surface.	It can simultaneously achieve morphological analysis and mechanical property testing.	It can only analyze the surface information of the sample, and the testing field is small.	~1 nm

**Table 5:** Customized structure design strategies in special application scenarios.

Application Scenarios	Failure Mechanism	Key Performance Requirements	Corresponding Structural Design Strategies
Fast charging	1. The drastic volume changes lead to an irreversible collapse of the conductive network. 2. Silicon particles detach from the conductive network, forming “dead Si”. 3. Significantly enhanced concentration polarization and electrochemical polarization. 4. The increased risk of lithium extraction.	1. Fast reaction kinetics in high-rate. 2. Outstanding structural stability and pulverization resistance. 3. Low interfacial resistance and stable SEI.	Nanostructure, hollow structure, three-dimensional conductive network structure.
Low temperatures	1. The restricted diffusion of Li^+^ in SEI, and the significantly increased charge transfer impedance and interfacial resistance. 2. The intensified concentration gradient. 3. Unsynchronized lithiation inside the particles induces stress concentration and electrode cracking.	1. The rapid transport capability of Li^+^ at low temperatures 2. The uniform lithiation of the entire electrode. 3. SEI with ionic conductivity, toughness and low-temperature stability	Nanostructure, carbon material composite structure.
Wide temperature range	1. Strategies optimized for a single temperature region degrade the performance in other temperature ranges. 2. The insufficient temperature adaptability of SEI. 3. The mismatch of thermal-physical-chemical in multicomponent electrode system.	1. Controllable and moderate volume expansion. 2. The temperature-adaptive SEI. 3. Thermodynamic, mechanical and electrochemical temperature compatibility	1. Construct three-dimensional structures via compounding with conductive and rigid skeletal materials. 2. Yolk core–shell structure.

**Table 6:** The comparative analysis of various preparation methods in terms of preparation cost, safety and salability.

Methods	Preparation Cost	Safety	Scalability Evaluation
Mechanical milling methods	Low production cost. 1. The raw material is inexpensive industrial silicon powder. 2. The post-treatment only mainly involves drying and screening.	Medium level of safety. Flammable silicon powder.	High scalability. The ball milling equipment is a mature device that supports continuous large-scale production.
Spray drying method	Medium synthesis cost. 1. Preparing the raw material into a solution requires the use of organic solvents. 2. The drying process needs high-temperature hot air.	Medium security level. 1. The volatility and flammability of organic solvents 2. The high temperature of the drying tower.	High scalability. 1. The spray drying equipment is a continuous production line. 2. The atomization and drying parameters are easy to adjust, and the product uniformity is high.
Chemical vapor method	High synthesis cost. 1. The silicon source requires high purity and is expensive. 2. Large energy consumption during high-temperature reactions.	Low safety. 1. The flammable, explosive and highly toxic silicon source. 2. The reaction process produces corrosive gases like HCl.	Moderate scalability. 1. Most CVD equipment is for batch production. 2. The temperature and silicon source concentration distribution within the equipment are uneven.
Template method	High production costs. 1. The template reagents and etching agents are expensive. 2. The raw material utilization rate is less than 50%.	Low safety. 1. The toxic and corrosive etchant. 2. During the high-temperature baking of the template, powder splashing is prone to occur.	Low scalability. 1. The template method involves non-continuous reactions 2. The complex template removal process.
Hydrothermal method	Moderate synthesis cost. 1. Low raw material prices, high raw material utilization rate and simple maintenance. 2. Medium equipment costs and moderate reaction energy consumption.	Medium safety level. The hydrothermal autoclave is prone to exploding under high temperature and high pressure.	Low scalability. Due to the size and safety requirements of the autoclave, it is impossible to achieve large-scale production, resulting in low production efficiency.
Magnesium thermal reduction method	Low preparation cost. 1. The raw material prices are extremely low, and the energy consumption is moderate. 2. The post-processing mainly involves acid washing, and the cost of consumables is low.	Low safety. 1. Magnesium powder is flammable. 2. The exothermic reaction caused by the magnesium thermal reduction releases a large amount of heat	Moderate scalability. During large-scale production, there may be issues of insufficient or excessive reduction due to the decrease in temperature uniformity.
Sol–gel method	High preparation cost. 1. The price of the organic silicon source is high and it requires high purity. 2. A large amount of organic solvents is used in the reaction process.	Medium safety level. 1. The organic solvents are flammable and volatile. 2. During the reaction process, there is a high-temperature reaction that may cause powder splashing.	Low scalability. The sol–gel process is a non-continuous process. When preparing in large quantities, the products are prone to agglomeration and structural defects.

**Table 7:** Electrochemical performance of silicon-based anodes in full-cells matched with different cathodes.

Full-Cell	Anode Materials	Cathode Materials	Electrode Loading (mg cm^−2^) (Up: Anode, Down: Cathode)	Areal Capacity (mAh cm^−2^) (Up: Anode, Down: Cathode)	Current Density (A g^−1^)	ICE	Cycle Number	Capacity Retention	Ref.
Si@TiSi_2_@NC‖NCM622	Silicon-carbon, TiSi_2_	LiNi_0.6_Co_0.2_Mn_0.2_O_2_	1.2	—	0.6	85.3%	50	80.2%	[105]
			12.1	—					
n-Si@G-C‖NCM811	Silicon-carbon	LiNi_0.8_Co_0.1_Mn_0.1_O_2_	4.3	2.42	1.5	—	300	71.3%	[41]
			8.2	2.2					
Si-CNTs‖NMC	Silicon-carbon	LiNi_0.8_Mn_0.1_Co_0.1_O_2_	6.13	7.65	0.16	85.1%	100	56%	[140]
			35	6.98					
Si NWs‖LMO	Silicon	LiMn_2_O_4_	1.04	2.09	0.12	—	500	42.3%	[88]
			—	1.89					
Si-CNT@PC‖LNMO	Silicon-carbon	LiNi_0.5_Mn_1.5_O_4_	0.42	0.2	1	72%	50	95%	[141]
			4.2	—					
Si NR‖LiCoO_2_	Silicon	LiCoO_2_	0.5	2	1	—	500	78%	[142]
			—	1.8					
GS@Si@C‖LiFeO_4_	Silicon-carbon	LiFeO_4_	1	1.48	0.5	82%	100	85.9%	[97]
			—	1.23					
MWCNT-wrapped SiP_2_‖LiFePO_4_	Silicon phosphide-carbon	LiFeO_4_	1.5	1.45	4	—	150	94%	[74]
			—	1.2					
SiN‖NCM622	Silicon nitride	LiNi_0.6_Co_0.2_Mn_0.2_O_2_	12.6	5.67	—	—	150	87%	[143]
			12.5	2.32					

## Data Availability

No new data were created or analyzed in this study. Data sharing is not applicable to this work.
